# MicroRNA-7 as a potential therapeutic target for aberrant NF-κB-driven distant metastasis of gastric cancer

**DOI:** 10.1186/s13046-019-1074-6

**Published:** 2019-02-06

**Authors:** Tingbo Ye, Meihua Yang, Daochao Huang, Xin Wang, Bingqian Xue, Na Tian, Xiaohui Xu, Liming Bao, Huajian Hu, Tiewei Lv, Yi Huang

**Affiliations:** 1 Chongqing key Laboratory of Child Infection and Immunity, Chongqing key Laboratory of Pediatric, Ministry of Education Key Laboratory of Child Development and Disorders, China International Science and Technology cooperation base of Child development and Critical disorders, Children’s Hospital of Chongqing Medical University, No.136 Zhongshan Erd Road, Yuzhong District, Chongqing, 400014 China; 2 Department of Neurosurgery, Xinqiao Hospital of Army Medical University, Chongqing, 400037 People’s Republic of China; 3 Department of Pathology, University of Colorado School of Medicine, Aurora, CO 80045 USA

**Keywords:** MiR-7, Gastric Cancer, Clinical outcome, NF-κB, Distant metastasis

## Abstract

**Background:**

Dysregulated miR-7 and aberrant NF-κB activation were reported in various human cancers. However, the expression profile, clinical relevance and dysregulated mechanism of miR-7 and NF-κB RelA/p65 in human gastric cancers (GC) metastasis remain largely unknown. This study is to investigate the expression profile, clinical relevance and dysregulated mechanism of miR-7 and NF-κB RelA/p65 in GC and to explore the potential therapeutic effect of miR-7 to GC distant metastasis.

**Methods:**

TCGA STAD and NCBI GEO database were used to investigate the expression profile of miR-7 and NF-κB RelA/p65 and clinical relevance. Lentivirus-mediated gene delivery was applied to explore the therapeutic effect of miR-7 in GC. Real-time PCR, FACS, IHC, IF, reporter gene assay, IP, pre-miRNA-7 processing and binding assays were performed.

**Results:**

Low miR-7 correlated with high RelA/p65 in GC with a clinical relevance that low miR-7 and high RelA/p65 as prognostic indicators of poor survival outcome of GC patients. Moreover, an impaired pre-miR-7 processing caused by dysregulated Dicer1 expression is associated with downregulated miR-7 in GC cells. Functionally, delivery of miR-7 displays therapeutic effects to GC lung and liver metastasis by alleviating hemangiogenesis, lymphangiogenesis as well as inflammation cells infiltration. Mechanistically, miR-7 suppresses NF-κB transcriptional activity and its downstream metastasis-related molecules Vimentin, ICAM-1, VCAM-1, MMP-2, MMP-9 and VEGF by reducing p65 and p-p65-ser536 expression. Pharmacologic prevention of NF-κB activator LPS obviously restored miR-7-suppressed NF-κB transcriptional activation and significantly reverted miR-7-inhibited cell migration and invasion.

**Conclusions:**

Our data suggest loss of miR-7 in GC promotes p65-mediated aberrant NF-κB activation, facilitating GC metastasis and ultimately resulting in the worse clinical outcome. Thus, miR-7 may act as novel prognostic biomarker and potential therapeutic target for aberrant NF-κB-driven GC distant metastasis.

**Electronic supplementary material:**

The online version of this article (10.1186/s13046-019-1074-6) contains supplementary material, which is available to authorized users.

## Introduction

Gastric cancer (GC) is the fourth leading cause of cancer-related death worldwide [1, 2]. Despite accelerated progress in diagnosis and treatments, the biological and molecular mechanisms underlying GC development are still incompletely understood. MicroRNAs (miRNAs, miRs) are small noncoding RNAs that act as post-transcriptional repressors of cancer-related genes through binding to the 3’-UTR of target mRNAs and thereby function as oncogenes or tumor suppressor genes [3]. Recent active research identified a series of dysregulated miRNAs that are involved in GC progression as oncogenes and tumor suppressors [4–7]. miRNA-7(miR-7), one of dysregulated miRNAs, has been characterized as a potential tumor suppressor in various cancers [7–14]. The latest study also reported the tumor suppressive role of miR-7 in GC tumorigenesis [12]. However, previous findings also implied an ambiguous role of miR-7 in regulating the complex network of oncogenes and tumor suppressors in different tumor types [12]. Therefore, it is necessary to revaluate miR-7 as potential biomarker or therapeutic target in the diagnosis and treatment of GC.

GC metastasis is the main cause of death in advanced GC patients [15, 16]. Aberrant NF-κB activation has been found to be frequently activated in most of GC and was regarded to be major contributor in the GC metastasis [17–20]. Our previous study firstly identified that GRIM-19, a mitochondrial inner membrane protein, was severely depressed in GC and that its loss contributed to GC tumorigenesis partly via STAT3 pathway [15]. In addition, we further demonstrated that delivery of GRIM-19 suppressed GC metastasis via blocking NF-κB-p65 activation [16, 17]. However, mechanisms underlying how GRIM-19 regulated NF-κB activation remains poorly understood. Interestingly, our unpublished data from miRNA microarray identified that miR-7 is one of GRIM-19-induced miRNA, indicating that miR-7 plays a critical role in anti-metastasis activation of GRIM-19. However, the expression profile and clinical relevance of miR-7 and NF-κB activation in GC metastasis remains largely unknown and the mechanism underlying dysregulated miR-7 in GC is yet to be elucidated. Moreover, much less information is available for the therapeutic effect of miR-7 in GC metastasis, especially in distant metastasis. Therefore, improved understanding of the role of miR-7 in GC metastasis should open new avenues and will develop novel therapeutic strategies for GC.

Thus, this study is aim to understand the expression profile, clinical relevance and dysregulated mechanism of miR-7 and RelA/p65 in GC progression and to explore whether and how delivering miR-7 could display therapeutic effect to GC metastasis.

## Materials and methods

### TCGA and NCBI GEO data analysis

To recapitulate the expression levels and clinical relevance of miR-7 and RelA/p65 in GC, data were obtained from the TCGA stomach adenocarcinoma dataset (STAD) and the NCBI GEO database. TCGA RNA-seq data were analyzed by first replacing all RSEM values, and then a log2 transformation was applied. Survival analysis including overall survival (OS), fist-progression survival (FPS), post-progression survival (PPS), progression-free survival (PFS) and distant metastasis-free survival (DMFS) were produced by the Kaplan-Meier survival analysis.

### Cell lines and reagents

The normal gastric epithelial cell line GES-1, human embryonic kidney HEK-293 cells, five human GC cell lines SGC-7901, BGC-823, HGC-27, MKN-28 and MKN-45 were obtained from the Cell Bank of the Chinese Academy of Science (Shanghai, China) or the American Type Culture Collection (ATCC, Manassas, VA, USA). Cells were cultured in DMEM or RPMI 1640 (Gibco BRL, Rockville, MD, USA) supplemented with 10% fetal bovine serum, 100 U/ml penicillin and 100 μg/ml streptomycin at 37 °C in a humidified chamber containing 5% CO_2_ as previously described [12, 16]. All chemical reagents were purchased from Sigma-Aldrich (St. Louis, MO, USA) unless otherwise indicated.

### RNA extraction and quantitative reverse transcription PCR (qRT-PCR)

Total RNA from cells was isolated using Tripure Isolation Reagent (Roche, Mannheim, Germany) according to the manufacturer’s instructions. QRT-PCR was performed to measure the expression of mature miR-7 using Bulge-Loop miRNA qRT-PCR Primer (RIOBOBIO, Guangzhou, China) and RNA-direct Realtime PCR Master Mix (TOYOBO, OSAKA, Japan) on CFX Connect Real-Time system (Bio-Rad). U6 RNA was used for endogenous control. Pri-miR-7 isoforms (pri-miR-7-1,2,3) and pre-miR-7 isoforms (pre-miR-7-1,2,3) were analyzed using modified real-time PCR in indicated cells as previously described [7]. Human18s rRNA was used as endogenous control. mRNA expressions of target genes were detected by qRT-PCR as our previously described [16]. The relative gene expression levels were normalized to those of β-actin and calculated using the 2^-ΔΔCt^ method. The primers sequences of target genes are listed in Additional file 1: Table S1.

### Cloning, in vitro transcription and labeling of pre-miR-7-1, immunoprecipitation of Dicer1 containing IP complex, in vitro pre-miR-7-1 processing assay and pre-miR-7-1 binding assay

The details were shown in Additional file 2: Supplemental Materials & Methods and Additional file 1: Table S3.

### Recombinant lentiviruses and transfection

Lentiviruses harboring mature miR-7 (LV-hsa-miR-7) were generated by transfection of 293 T cells with the GV217 (Ubi-EGFP-MCS) expression construct (http://www.genechem.com.cn:8080/Pro_show.aspx?plb=630), and the final recombined and control lentiviruses (LV-control) were purchased from GeneChem Co, Shanghai, China. HGC-27 and MKN-28 Cells at 60% confluence were infected with a multiplicity of infection (MOI) at 10 and 20, respectively. The infected GFP^+^ cells were sorted by flow cytometry (FACS Aria ǁ, BD) and were expanded in vitro to establish stable cell lines as previously described [16].

### Cell viability, cell cycle, colony formation assay and apoptosis analysis

Cell viability was measured using the enhanced Cell Counting Kit-8 (CCK-8) (Beyotime, Jiangsu, China) according to the manufacturer’s instructions. Cells were seeded into 96-well plates at a density of 1000 cells per well as indicated time point. Absorbance at 450 nm was measured using a Synergy H1 microplate reader (Bio Tek). Eight hundred per well of indicated cells were seeded in 6-well plates. Cell colonies were stained with crystal violet and counted after 3 weeks of culture. Cell cycle and cell apoptosis were analyzed using flow cytometry (FACS Calibur, BD) as previously described [15]. Cells apoptosis were identified using Annexin V-PE/7-AAD apoptosis Kit (KeyGEN Biotech, Nanjing, China) according to the manufacturer’s instructions. The data were analyzed with FlowJo software (Tree Star, Ashland, OR).

### Cell migration and cell invasion assays

Cell migration and cell invasion assays were performed in 24-well non-coated or Matrigel-coated Transwell chambers (8-μm pore size, Corning, NY, USA) as described previously [16]. 1 × 10^5^ cells were plated in the top chamber with 200 μl of serum-free medium, and 800 μl medium supplemented with 20% FBS was used as a chemoattractant in the bottom chamber. Cells were then incubated for 24 h to allow for migration or invasion and were fixed and stained with Crystal Violet Staining Solution (Beyotime, Jiangsu, China). The images of the migrated or invaded cells were captured, and the number of cells was counted in 5–10 random fields for each group and summarized as mean ± standard deviation (SD) for statistical analysis. For LPS intervention assay, 1 × 10^5^ miR-7 expressing cells were treated with or without LPS (50 ng/ml or 100 ng/ml) and cell migration and cell invasion assays were performed as above described.

### Flow cytometry analysis

The protein expression in GC cells was determined by flow cytometry using indirect immunofluorescence staining with anti-human specific antibodies as described previously [21]. All antibodies are listed in Additional file 1: Table S2. All stained cells were analyzed on a FACS Calibur flow cytometer (BD Bioscience) and data analyzed with FlowJo software (Tree Star, Ashland, OR).

### Immunofluorescence analysis

Immunofluorescence analysis were performed as previously described [15]. Cells were fixed with 4% paraformaldehyde for 20 min at room temperature and blocked with QuickBlock™ Blocking Buffer (Beyotime, Jiangsu, China) for 15 min at room temperature. Then cells were incubated with primary antibodies at 4 °C overnight, followed by incubation for 1 h at room temperature with AF555 or AF647-conjugated secondary antibody (Bioss, Beijing, China). Nuclei were counterstained with 4′,6- diamidino-2-phenylindole (DAPI). Images were captured using a confocal microscope (Zeiss, Jena, Germany).

### Luciferase reporter assay

Cells were seeded in 96-well plates at approximately 1 × 10^4^ cells per well. Cells were transfected with the pNF-κB-TA-Luc reporter plasmid (90 ng) (Beyotime Inc., Haimen, China) using Lipofectamine 2000 (Thermo Fisher Scientific, MA, USA). pRL-TK *Reniila* plasmid (10 ng) (Promega, Madison, USA) was co-transfected to normalize transfection efficiency. After another 24 h incubation, the Firefly and Renilla luciferase activities were quantified using the Dual-Luciferase® Reporter Assay System (Promega, Madison, USA). Relative luciferase (Luc) activity were calculated by the ratio of Firefly and Renilla luciferase signals. For LPS intervention assay, 1 × 10^4^ indicated cells were transfected with reporter plasmids for 24 h and then treated with or without LPS (50 ng/ml or 100 ng/ml) and luciferase activity were detected as described above.

### In vivo experimental metastasis mice models and overall survival assay

BALB/C nude (nu/nu) mice (6–8 weeks, Female, SPF degree, 20 ± 3 g) were purchased from Laboratory Animal Center of Chongqing medical University (Chongqing, China). All procedures were approved by the Institutional Animal Care Committee. Experimental distant metastasis models were established by tail vein (TV)-injection with lentivirus transfected HGC-27 and MKN-28 cells (2 × 10^6^ cells/100 μl PBS /mouse, *n* = 8 mice/group), respectively. Post-TV injection, body weight and the status of nude mice were monitored every 3 days. At the end of experiments, experimental mice were sacrificed and liver & lung tissues were harvested for the analysis of H&E and Immunohistochemistry staining. For the overall survival assay, the experimental metastasis mice model was established using the above method as in vivo experimental metastasis mice models (*n* = 10 mice/group), the observation period of overall survival was up to 70 days until all experimental mice died. Survival curves were generated according to the Kaplan-Meier method.

### H&E staining and immunohistochemistry

H&E staining and Immunohistochemistry (IHC) were performed as described previously [15, 22]. Briefly, metastatic tissues from indicated groups were fixed in 10% buffered formalin and embedded in paraffin. Tissue sections (4 μm) were subjected to H&E staining. IHC staining was performed to analyzed using Elivision plus Polyer HRP IHC Kit (Maixin, Fujian, China) and DAB kit (ZSGB-Bio, Beijing, China). All antibodies for IHC used are listed in Additional file 1: Table S2.

### Statistics

Statistical calculations were carried out with the GraphPad Prism 6 (GraphPad Software) as previously described [15]. All data were expressed as means ± SD. Two-paired Student *t* test was used to analyze data from cell growth, foci formation, and tumor metastasis in nude mice and protein relative expression analysis. Survival curves were generated according to the Kaplan-Meier method and the statistical analysis was done by log-rank test. The value of *p* < 0.05 was considered statistically significant.

## Results

### Downregulated miR-7 is correlated with high RelA/p65 in GC

**Fig. 1:**
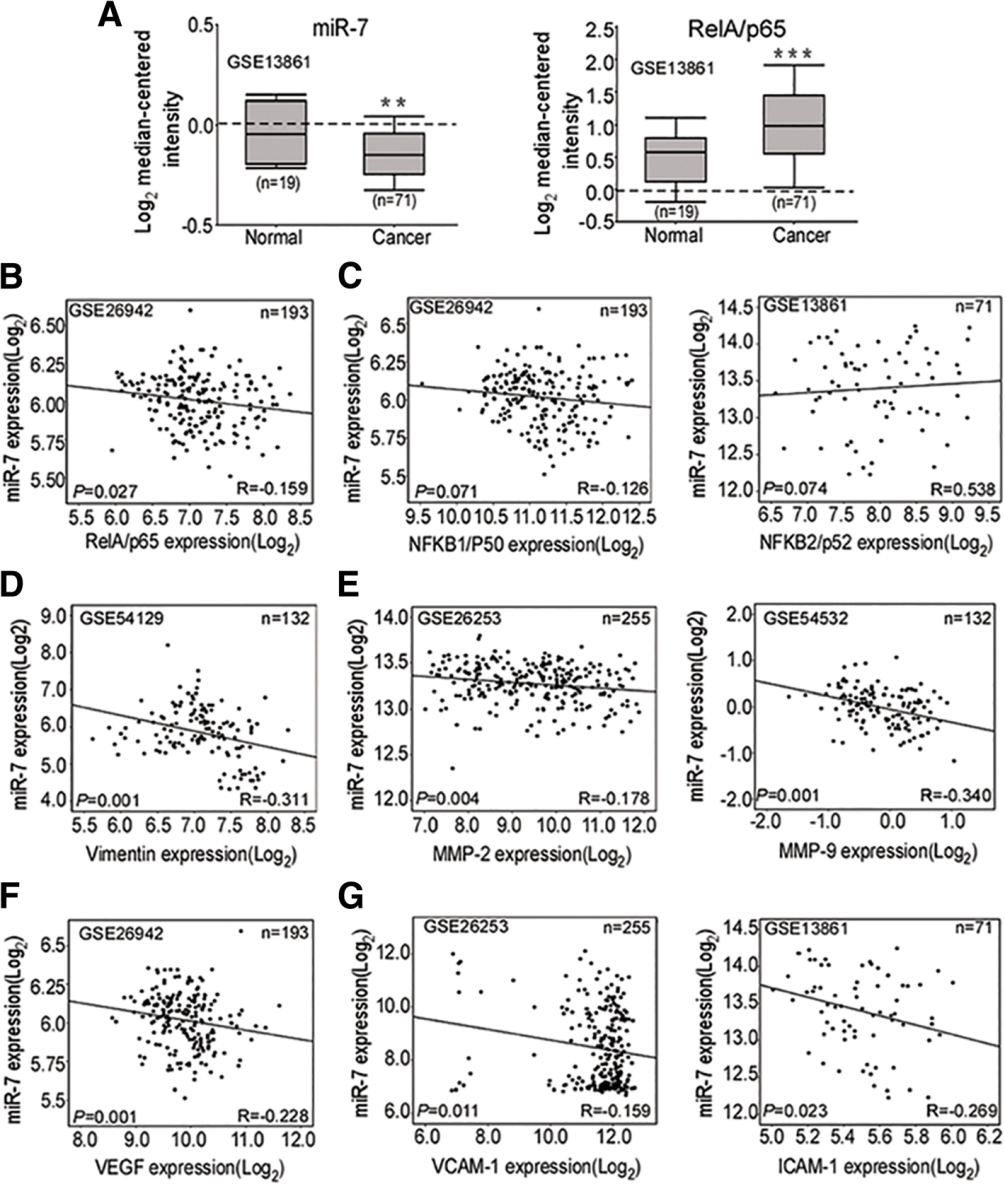
Downregulated miR-7 is correlated with high RelA/p65 in GC. **a** miR-7 is downregulated whereas RelA/p65 mRNA is increased in GC. Expression of miR-7 and RelA/p65 mRNA was analyzed using the same human GC cohort from the NCBI/GEO/GSE13861 (Normal: normal gastric mucosa; Cancer: gastric cancer). The box plot indicated the log2 transformed mRNA median gene expression level in the tissues. **b** Correlation of miR-7 with RelA/p65 mRNA expression (*n* = 193, r = − 0.159, *P* = 0.027) in GC from NCBI/GEO/GSE26942. **c** Expression correlation between miR-7 and NFKB1/p50 (*n* = 193, r = − 0.126, *P* = 0.071, left panel), NFKB2/p52 mRNA expression (*n* = 71, r = 0.538, *P* = 0.074; right panel) in GC from NCBI/GEO /GSE26942 and GSE13861. **d** Expression correlation between miR-7 and Vimentin (*n* = 132, r = − 0.311, *P* = 0.001) mRNA expression in GC from NCBI/GEO/ GSE54129. **e-g** Expression correlation between miR-7 and MMP-2 (*n* = 255, *r* = − 0178, *P* = 0.001; **e**, left panel), MMP-9 (*n* = 132, *r* = − 0.340, *P* = 0.001; **e**, right panel), VEGF (*n* = 193,*r* = − 0.228, *P* = 0.001; **f**, VCAM-1 (*n* = 255, left panel, *r* = − 0.159, *P* = 0.011; **g**, left panel) and ICAM-1(*n* = 71,*r* = − 0.263, *P* = 0.023; **g** right panel,) mRNA expression respectively according to NCBI GEO database(GSE26253, GSE54532, GSE26942, GSE13861). The expression correlation was determined with Pearson’s correlation analysis. ** P < 0.01; *** P < 0.001

To investigate the expression profile of miR-7 and RelA/p65 in GC, we evaluate expression of miR-7, RelA/p65 and NF-kB downstream metastatic targets from TCGA STAD and NCBI GEO database. We found low miR-7 and high RelA/p65 expressions in the primary gastric tumors compared with non-tumorous tissues (Fig. 1a). Meanwhile, mRNA levels of NF-kB downstream metastasis-related genes including Vimentin, MMP-2, MMP-9, VEGF, ICAM-1 and VCAM-1 were significantly enhanced in the primary gastric tumors (Additional file 3: Figure S1). To elucidate correlation of miR-7 and NF-κB subunits, we performed gene correlation analysis from NCBI GEO data sets and showed that low miR-7 was significantly correlated with RelA/p65 mRNA expression (Fig. 1b) instead of NFKB1/p50 and NFKB2/p52 (Fig. 1c). Moreover, we further investigated the correlation of miR-7 and NF-kB downstream metastatic targets. Consistently, miR-7 expression displayed a negative correlation with mRNA expression of Vimentin, MMP-2, MMP-9, VEGF, VCAM-1 and ICAM-1 (Fig. 1d-g). Thus, these results indicated that downregulated miR-7 might be associated with aberrant RelA/p65-mediated NF-kB.

### Low miR-7 and high RelA/p65 expressions predict poor prognosis of GC patients

**Fig. 2:**
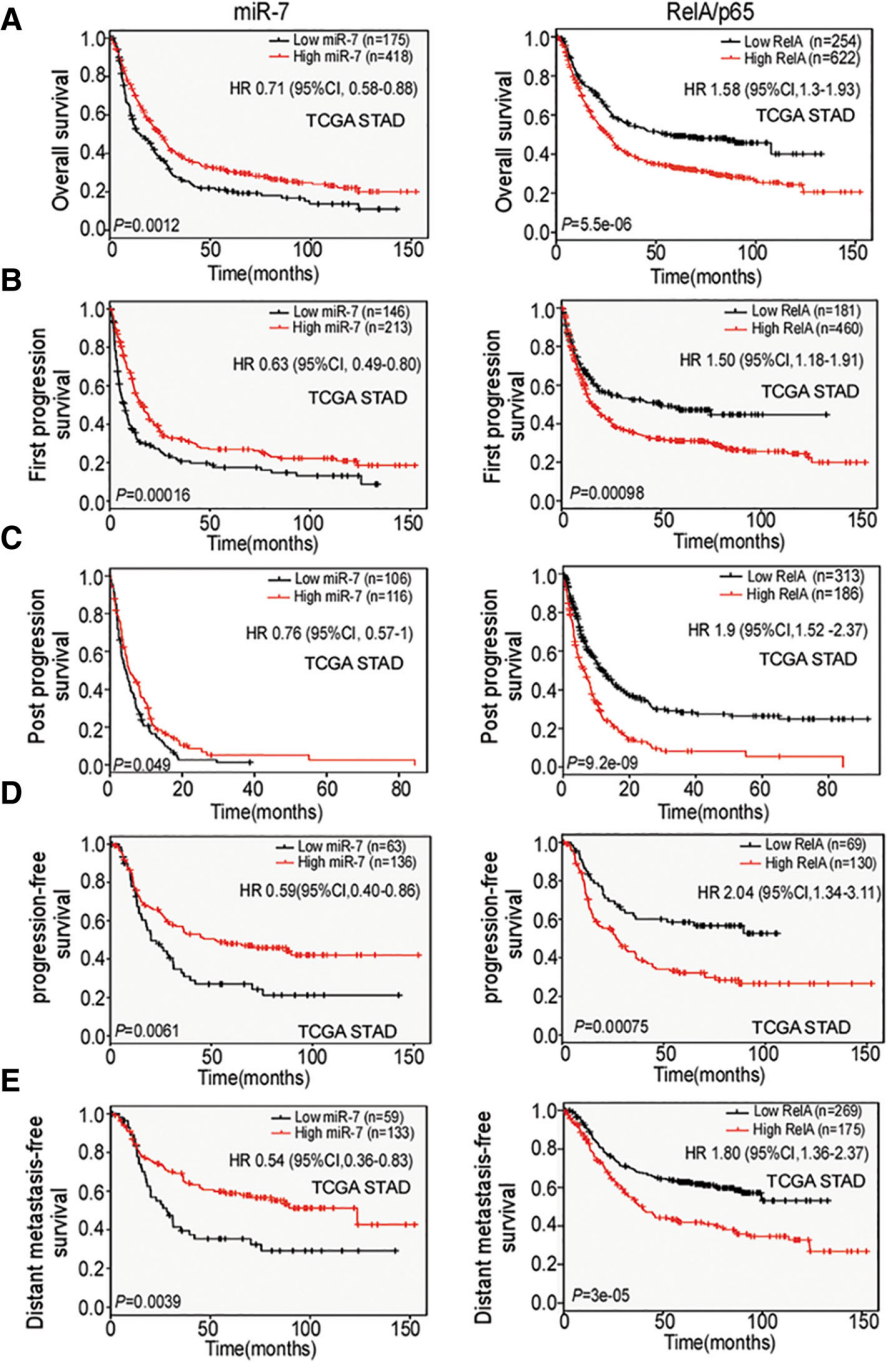
Low miR-7 and high RelA/p65 expression are correlated with poor prognosis of GC patients. The correlations between miR-7, RelA/p65 and multiply survival rates were determined by Kaplan-Meier analysis from TCGA STAD database. **a** miR-7 downregulation is related to shorter overall survival (OS) (*n* = 593, *P* = 0.0012, left panel). High RelA/p65 indicated poor OS (*n* = 876, *P* = 5.5e-06, right panel). **b** Low miR-7 indicated shorter fist-progression survival (FPS) (*n* = 359, *P* = 0.00016, left panel). High RelA/p65 indicated worse FPS (*n* = 641, *P* = 0.00098, right panel) according to TCGA cohort. **c** Low miR-7 indicated poor post-progression survival (PPS) (*n* = 222, *P* = 0.049, left panel). High RelA/p65 indicated shorter PPS (*n* = 499, *P* = 9.2e-09, right panel). **d** Low miR-7 is correlated with shorter progression-free survival (PFS) (*n* = 222, *P* = 0.049, left panel). High RelA/p65 indicated poor PFS (*n* = 499, P = 9.2e-09, right panel). **e** Low miR-7 is correlated with worse distant metastasis-free survival (DMFS) (*n* = 192, *P* = 0.0039, left panel). High RelA/p65 indicated shorter DMFS (*n* = 444, *P* = 3e-05, right panel). *P* values were determined by log-rank test

Improved survival rate of GC patients has been considered the most important therapeutic objective in advanced GC cancer. To investigate the prognostic and potentially predictive value of miR-7 and RelA/p65 in GC, we conducted a serial of survival analysis for OS, FPS, PPS, PFS and DMFS through TCGA STAD database. Survival rate analysis showed that miR-7 expression was positively whereas RelA/p65 was negatively correlated with OS, FPS, PPS, PFS and DMFS (Fig. 2a-e) among GC patients in TCGA cohort. These results suggest that miR-7 and RelA/p65 levels are potential prognostic biomarkers for GC patients.

### Downregulation of miR-7 with a mechanism involving in an impaired mature processing of miR-7 biogenesis in GC cells

**Fig. 3:**
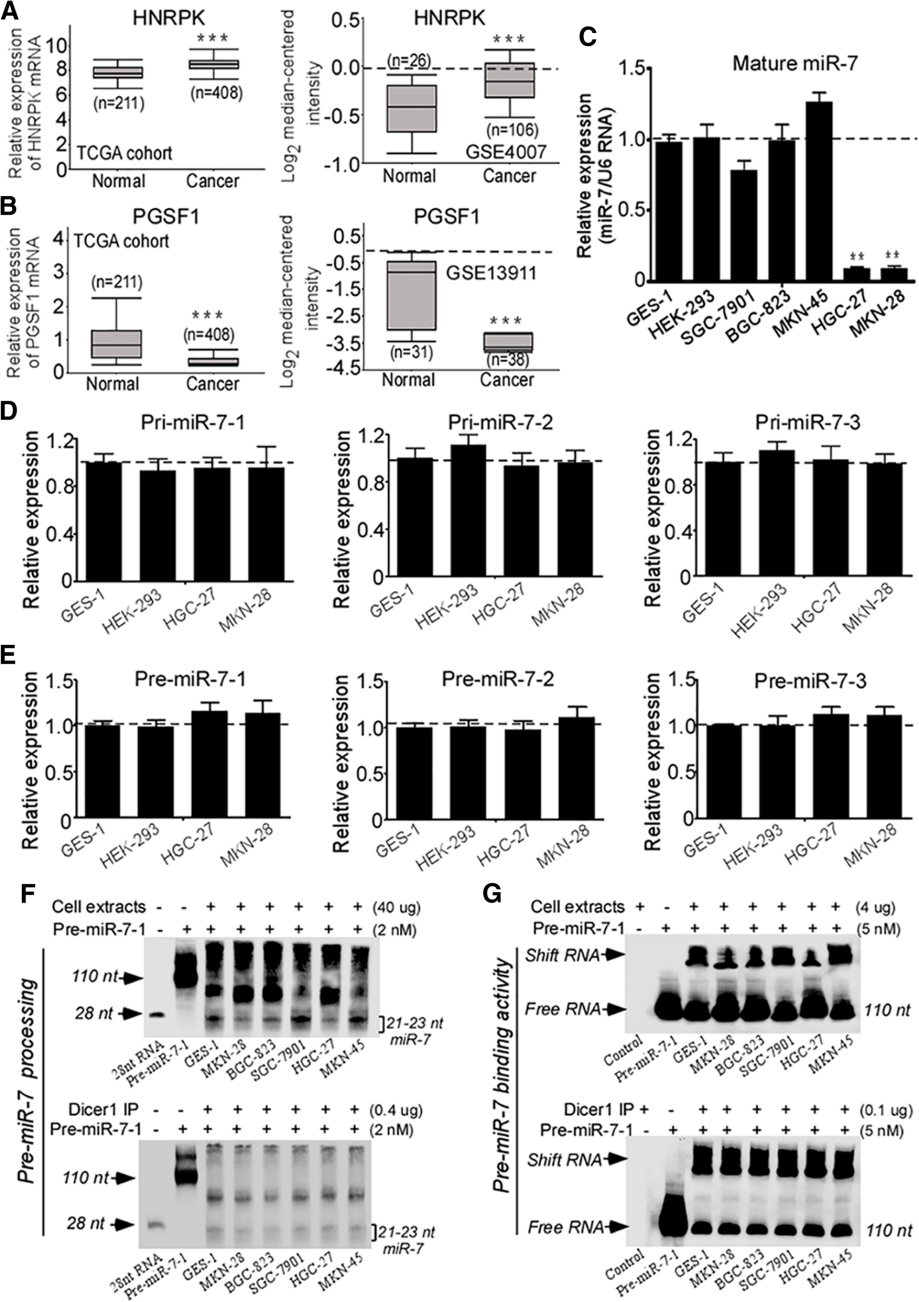
Impaired pre-miR-7 processing is responsible for downregulated miR-7 in GC cells. **a** Upregulated HNRPK (miR-7-1 host gene) mRNA expression in TCGA STAD (*n* = 619, *P* < 0.001) and in the NCBI/GEO/GSE4007 database l (*n* = 132, *P* < 0.001). **b** Downregulated PGSF1 (miR-7-3 host gene) mRNA expression of GC in TCGA STAD (*n* = 619, *P* < 0.001) and in the NCBI/GEO/GSE13911 database (*n* = 69, *P* < 0.001). **c** Expression of mature miR-7 in five GC cell lines relative to GES-1 and HEK-293 cells. Total RNA was extracted from indicated cells. Expression of mature miR-7 was detected by real-time PCR. U6 RNA was used as an internal control. **d-e** Expression of pri-miR-7 and pre-miR-7 isoforms in GC cells. Pri-miR-7 isoforms (pri-miR-7-1,2,3) (**d**) and pre-miR-7 isoforms (pre-miR-7-1,2,3) (**e**) were quantitated by modified real-time PCR in HGC-27 and MKN-28 cells compared with GES-1 and HEK-293 cells. Human 18S rRNA was used as an internal control. **f** Pre-miR-7-1 processing assay. Pre-miR-7-1 RNA (2 nM) was incubated in processing buffer with cell extracts (Uper panel) or Dicer1 containing IP complex (Lower panel) respectively. The 110 nt pre-miR-7-1 and mature ~ 21-23 nt miR-7 were detected by RNA gel blot after processing action. **g** Pre-miR-7-1 binding assay. Pre-miR-7-1 RNA (5 nM) was incubated in binding buffer with cell extracts (Uper panel) or Dicer1 containing IP complex (Lower panel) respectively. RNA gel blot was used to detect shifted binding pre-miR-7-1 RNA and free pre-miR-7-1 RNA (110 nt) after binding action. Data are presented as mean ± SD of three independent experiments. * P < 0.05; ** P < 0.01 between the indicated two groups determined by paired student’s *t* test

Mature miR-7 is individually transcribed and processed from 3 different gene locus in human genome. In these three miR-7 isoforms, miR-7-1 and miR-7-3 are located within introns of the HNRPK and PGSF1 genes, respectively, whereas miR-7-2 is intergenic [7]. Thus, we analyzed approximate host genes mRNA expression of the miR-7-1 and miR-7-3 in TCGA STAD database. HNRPK, the miR-7-1 host gene showed higher expression (Fig. 3a) whereas PGSF1, the host gene for miR-7-3, showed lower expression in GC versus normal tissues (Fig. 3b). These data showed that decreased transcription of the host gene was not responsible for the decreased miR-7 expression, at least for miR-7-1, implying that other mechanisms were responsible.

To further considerate mechanisms for the dysregulated miR-7 in human GC, we firstly quantitated mature miR-7 expression in five human GC cell lines (SGC-7901, BGC-823, MKN-45, HGC-27 and MKN-28) as well as human normal GES-1 and HEK-293 cells. Compared with GES-1 and HEK-293 cells, HGC-27 and MKN-28 cells exhibited a marked decrease of mature miR-7 whereas SGC-7901, BGC-823 and MKN-45 GC cell lines displayed no significant mature miR-7 difference (Fig. 3c), indicating mature miR-7 is dysregulated in HGC-27 and MKN-28 GC cell lines.

miR-7 biogenesis is usually controlled by three key processing: transcriptional processing of pri-miRNAs from genomic DNA, the processing of pri-miRNAs to pre-miRNAs and the mature processing from pre-miRNAs to miR-7. Since the expression profiles of pri-miR-7 s and pre-miR-7 s are absent in TCGA STAD database, we quantitated pri- and pre- forms of all three miR-7 isoforms in GC cells using modified real-time PCR. However, we did not observe significant alterations of pri-miR-7 s (pri-miR-7-1,2,3) and pre-miR-7 s (pre-miR-7-1,2,3) in HGC-27 and MKN-28 cells compared with GES-1 and HEK-293 cells (Fig. 3d-e). Meanwhile, we also observed relative higher or normal HNRPK levels but lower PGSF1 mRNA expressions in GC cells (Additional file 4: Figure S2A), whose expressive trends are similar with the results from TCGA STAD, further indicating that the host gene transcriptions were not responsible for the decreased miR-7 expression.

To obtain more mechanistic insights into the dysregulated miR-7 expression, we analyzed the mRNA levels of Drosha and Dicer1 enzymes, which are essential to pri-miRNA and pre-miRNA processing respectively. We found relative higher or normal Drosha levels but significant lower Dicer1 expressions in GC cells (Additional file 4: Figure S2B), suggested that dysregulated Dicer1 expression might contribute to the downregulation of miR-7 in GC cells. Therefore, we next conducted pre-miRNA-7 processing and binding assays to test Dicer1 enzyme activity using pre-miR-7-1. To this end, we generated 110-nt single stranded (ss) pre-miR-7-1 RNA as substrate by DNA cloning and T7 polymerase in vitro transcription and labeling (Additional file 5: Figure S3) and further performed these analysis using cell extracts or Dicer containing IP complex respectively. In cell extracts, we found that HGC-27 and MKN-28 cells displayed decreased processing abilities to product 21-23 nt mature miR-7 from 110 nt pre-miR-7-1 RNA and reduced in vitro binding activities to pre-miR-7-1 RNA compared with other cells (Fig. 3f-g, upper panel). Interestingly, using Dicer1 containing IP complex, we found that equal amount of Dicer1 IP complex from different cells extracts have similar processing and binding activities of pre-miR-7-1 RNA (Fig. 3f-g, lower panel). Therefore, these data strongly indicate that dysregulated Dicer1 expression impaired pre-miR-7 processing thereby contributed to downregulation of miR-7 expression in GC cells.

### Restoration of miR-7 decreases viability and invasiveness of GC cells

**Fig. 4:**
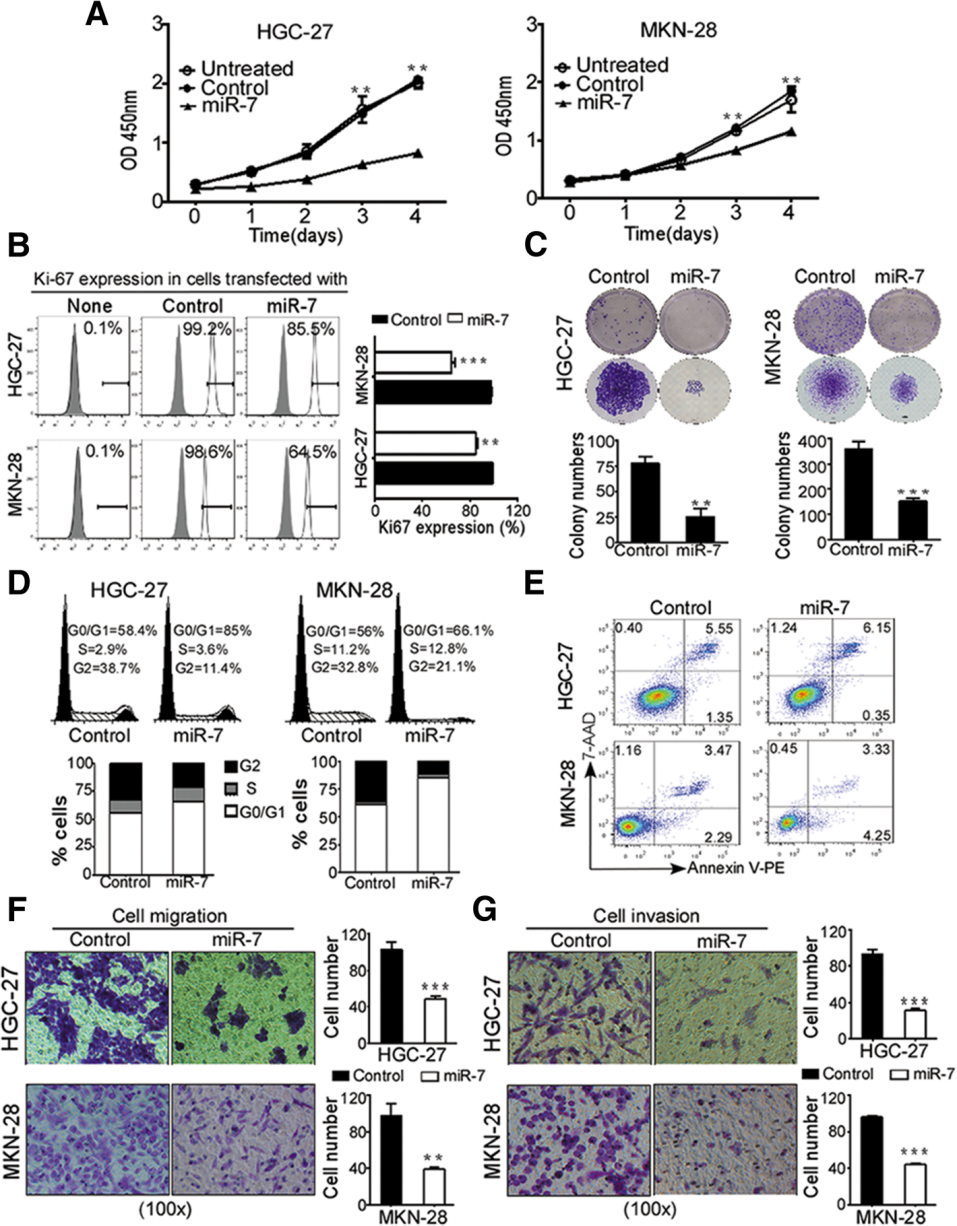
Restoration of miR-7 inhibits GC cell viability and invasiveness in vitro*.* HGC-27 and MKN-28 cells were stably transfected with mature miR-7 or negative control, respectively. **a** and **b** Restoration of miR-7 expression decreased Cell viability and inhibited Ki67 expression in GC cells. Cell viability (**a**) was determined by CCK-8 assay at absorbance 450 nm for 5 consecutive days. Ki67 protein expression in indicated cells (**b**) were measured by Flow cytometry using specific Ki67 antibodies. Representative images are shown. **c** Restoration of miR-7 expression suppressed Colony formation in GC cells. Colony formation assay was performed to examine colony formation ability in indicated cells. Colony numbers were counted for 3 weeks after transfection. Representative images of colonies formed are shown. Bar, 100 μm. **d** and **e** miR-7 expression promoted significant G0/G1 phase arrest but not cell apoptosis. Cell cycle were measured by flow cytometry analysis after PI staining in indicated cells (**d**). Cell apoptosis were detected by FACS analysis using Annexin V-PE/7-AAD double staining (**e**). Representative images of colonies formed and FACS analysis are shown. Data are presented as mean ± SD of three independent experiments. **f** and **g** Restoration of miR-7 inhibited GC cell migration and invasion. Cell migration assay (**f**) and cell invasion assay (**g**) were performed in 24-well non-coated or Matrigel-coated Transwell Chambers (8-μm pore size). Cell numbers were counted in 5–10 random fields. Data are presented as mean ± SD of three independent experiments. Representative images are shown. ** *p* < 0.01 and *** *p* < 0.001 between the indicated two groups determined by paired student’s *t* test

Given that miR-7 is downregulated in GC, we next investigated whether delivering mature miR-7 has therapeutic potential in GC. We generated lentiviral vectors harboring mature miR-7 (LV-miR-7) for the restoration of miR-7 in HGC-27 and MKN-28 cells (Additional file 6: Figure S4A-B). QRT-PCR showed that lentivirus-mediated miR-7 transfection stably restored exogenous miR-7 levels up to 2 folds upregulation compared with controls (Additional file 6: Figure S4C). CCK-8 assay showed a significant decrease of cell viability from day 3 following mature miR-7 transfection in both HGC-27 and MKN-28 cells (Fig. 4a). Cell proliferative activity was also confirmed by the Ki67 expression with FACS analysis (Fig. 4b) and IF staining (Additional file 7: Figure S5). Meanwhile, colony formation assay showed that miR-7 transfection significantly decreased colony numbers and diminished colony sizes compared with control groups (Fig. 4c). In parallel, cell cycle assay showed that miR-7 transfection induced a marked increase in G0/G1 fraction and a significant decrease in G2-M fraction but not obvious sub-G0/G1 population (Fig. 4d), indicating a typical G0/G1 cell cycle arrest. In addition, cell apoptosis analysis by Annexin V-PE/7-AAD staining showed no significant cell apoptosis or cell death (Fig. 4e). These results suggest that miR-7 suppressed GC cells viability by inducing cell cycle arrest but not cell apoptosis.

To further elucidate the role of miR-7 in metastasis in vitro, we assessed the impact of miR-7 on cell migration and cell invasion in vitro. Cell migration assay showed that miR-7 inhibited the ability to migrate across the transwell membrane in HGC-27 and MKN-28 cells (Fig. 4f). While, cell invasion assay showed that miR-7 expression markedly abrogated the ability of these cells to invade into the Matrigel-coated member (Fig. 4g). Collectively, these in vitro data demonstrate the capacity of miR-7 in suppressing GC metastasis in vitro.

### Delivering miR-7 inhibited GC distant metastasis and improved OS in vivo

**Fig. 5:**
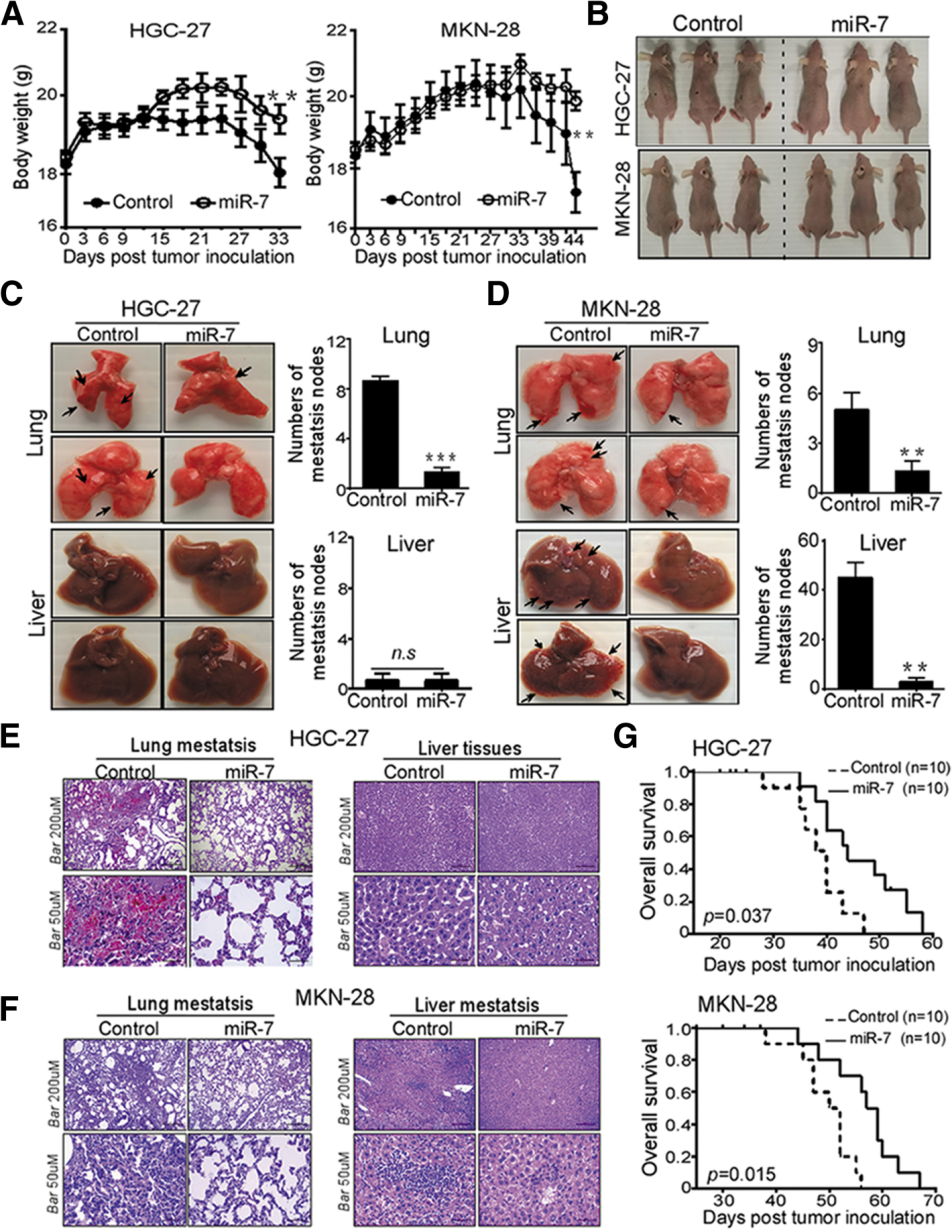
miR-7 inhibited GC distant metastasis and improved overall survival in vivo*.* HGC-27 and MKN-28 cells were stalely transfected with indicated Lentivirus (MiR-7 and Control). After sorting purification, cells (2 × 10^6^ /mice in 100 μl PBS buffer) were injected tail intravenously into the nu/nu mice (*n* = 8 mice/group). **a** and **b** Restoration of miR-7 in GC cells inhibited body weight loss in experimental metastasis mice. Post-transfection, body weight and the status of nude mice were monitored every 3 days and presented as mean ± SD (**a**). Representative images of the mice sizes are shown (**b**). **c-f** miR-7 markedly suppressed the lung and liver metastasis of GC cells. After indicated time point, lungs and livers from experimental mice were collected and metastatic tumor of the lung and liver surfaces were counted under a dissecting microscope (**c** and **d**) and were confirmed by H&E staining on sections from embedded lung and liver tissues (E and F). metastatic tumor was indicated by black arrow. Representative images of metastatic lung and liver tissues and H&E staining are shown. Scale bars indicate 50 μm and 200 μm. **g** miR-7 markedly improved overall survival (OS) in experimental metastasis mice. Kaplan-Meier analysis was used to analyze OS in established experimental metastasis mice (*n* = 10mice/group). Log-rank test was used for Kaplan-Meier survival analyses. Data are presented as mean ± SD. *p < 0.05, **p < 0.01 and ***p < 0.001 between the indicated two groups determined by paired student’s *t* test

Distant liver and lung metastasis are the most frequent forms of advanced GC metastasis [2]. We next performed complementary in vivo studies to explore anti- metastasis effects of miR-7 using experimental metastasis mice models by adoptive transfer of HGC-27 and MKN-28 cells expressing miR-7 or control. We found that body weight from mice injected with HGC-27 and MKN-28 cells expressing miR-7 was markedly decreased starting at day 33 and day 34 compared with corresponding controls (Fig. 5a-b). Subsequently, tumor metastasis was evaluated by analyzing metastatic nodes in lung and liver tissues at the end of these experiments. In HGC-27 injected mice, miR-7 restoration significantly decreased lung but not liver metastasis in vivo (Fig. 5c). Whereas a significantly decreased number of metastatic nodes was observed in both lung and liver tissues from MKN-28-injected mice models (Fig. 5d). Furthermore, H & E staining for embedded lung and liver tissues showed that high amount of tumor cells infiltrated into lungs and livers obtained from control groups but not from the miR-7 transfection group (Fig. 5e-f), suggesting that delivering miR-7 inhibited GC distant lung and liver metastasis in vivo.

Given that miR-7 is correlated with better OS in GC patients, we also assessed the effect of miR-7 on OS in metastatic mice models. As shown in Fig. 5g, delivery of miR-7 significantly prolonged OS of metastatic models from HGC-27 and MKN-28 cells, indicating that delivering miR-7 displays an ability to improve OS in metastasis mice models. These in vivo observations indicated that miR-7 may serve as a potential therapeutic tool for anti-metastasis of GC.

### VEGF driven-hemangiogenesis and lymphangiogenesis are involved in anti-metastasis activation of miR-7 in GC

**Fig. 6:**
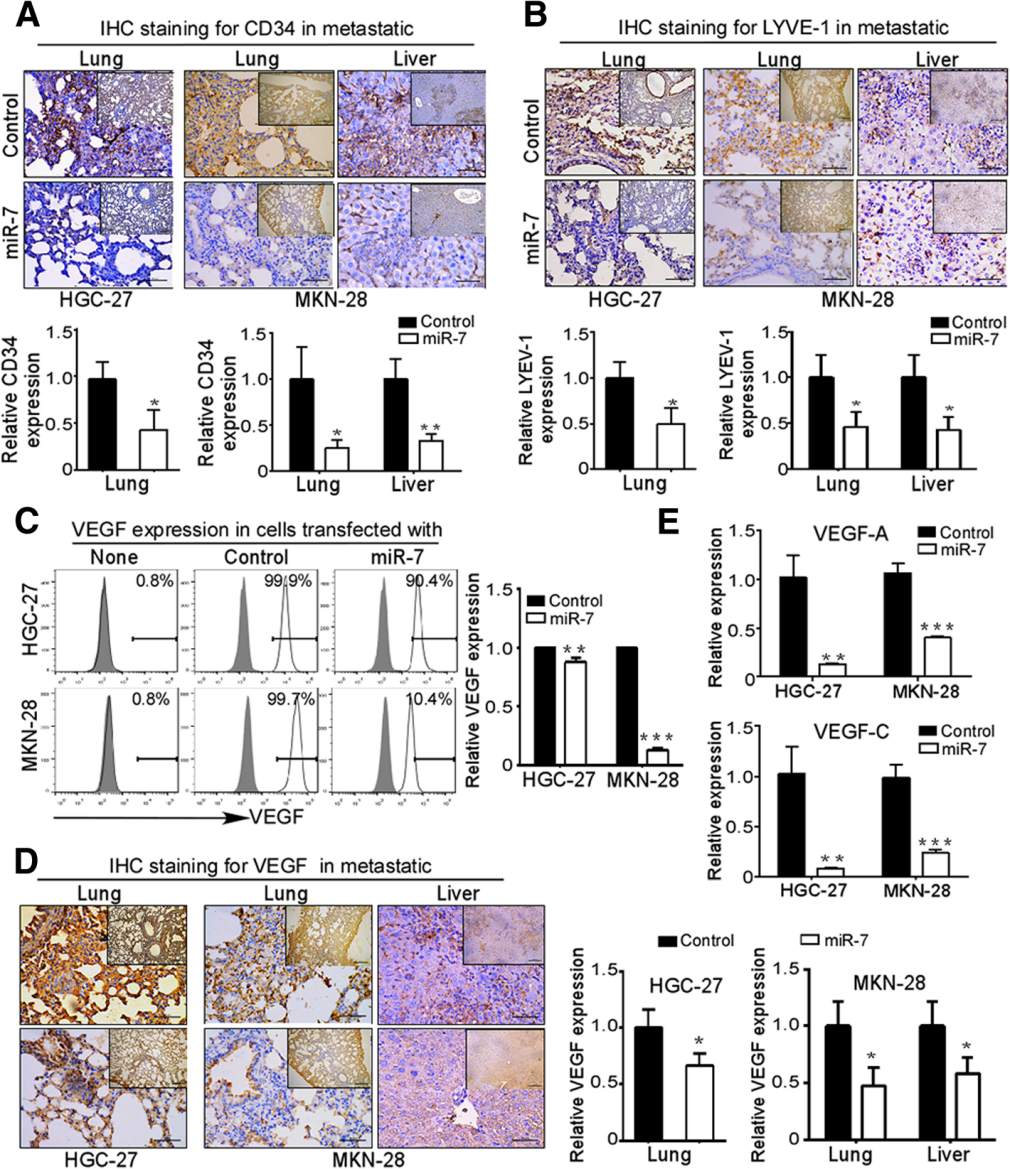
VEGF driven-hemangiogenesis and lymphangiogenesis are involved in anti-metastasis activation of miR-7 in GC. **a** and **b** miR-7 expression inhibited hemangiogenesis and lymphangiogenesis in vivo. Experimental metastasis mice from HGC-27 and MKN-28 cells were established and metastatic lung and liver tissues were collected as described in Materials & Methods. Hemangiogenesis was evaluated in metastatic lung and liver by IHC staining for CD34 hemendothelial marker (**a**). Lymphangiogenesis was evaluated by detecting Lymphatic vessels density through IHC staining for LYVE-1 (**b**). Representative IHC images are shown. Data are presented as mean ± SD. **c-d** miR-7 alleviated VEGF expression in vitro and in vivo. VEGF expression in GC cells were detected by flow cytometry (**c**). IHC staining was used to evaluate VEGF expression in metastatic lung and liver tissues (**d**). Representative IHC images are shown. **e** miR-7 alleviated VEGF-A and VEGF-C expression. VEGF-A and VEGF-C mRNA were detected by Real-Time PCR. Data are presented as mean ± SD. *p < 0.05, **p < 0.01, ***p < 0.001 between the indicated two groups determined by paired student’s *t* test. Scale bars: (main) 50 μm; (inset) 200 μm

Hemangiogenesis and lymphangiogenesis, two important initial steps, play crucial roles in tumor metastasis [18]. Therefore, we evaluated hemangiogenesis in metastatic lung and liver tissues by IHC staining using CD34 as an hemendothelial marker. As shown in Fig. 6a, a significant decreased MVD in metastatic lung and liver tissues of miR-7 transfected group as indicated by CD34 staining. Like hemangiogenesis, lymphangiogenesis has gained much attention for the increase of the risk for metastasis as an important initial step both in human tumors and animal models [18]. We found decreased LYVE-1 staining densities in metastatic lung and liver tissues of miR-7 groups compared with control groups (Fig. 6b). These results indicate a robust inhibition of hemangiogenesis and lymphangiogenesis by miR-7 in metastatic tissues.

To explore possible mechanisms, we explored the difference expression of VEGF, a critically major driver of hemangiogenesis and lymphangiogenesis during the tumor metastasis [18]. We found miR-7 transfection markedly reduced VEGF production as indicated by flow cytometry analysis (Fig. 6c) and IHC staining for metastatic tissues (Fig. 6d). Furthermore, we also distinguished the expression of VEGF isoforms by Real-time PCR for VEGF-A and VEGF-C and found that both VEGF-A and VEGF-C expressions were significantly downregulated in miR-7-transfected cells compared with control groups (Fig. 6e). Overall, these data show that miR-7 inhibits GC metastasis in vivo by suppressing hemangiogenesis and lymphangiogenesis via reducing VEGF-A and VEGF-C secretion.

### miR-7 improves inflammation cells infiltration in vivo

**Fig. 7:**
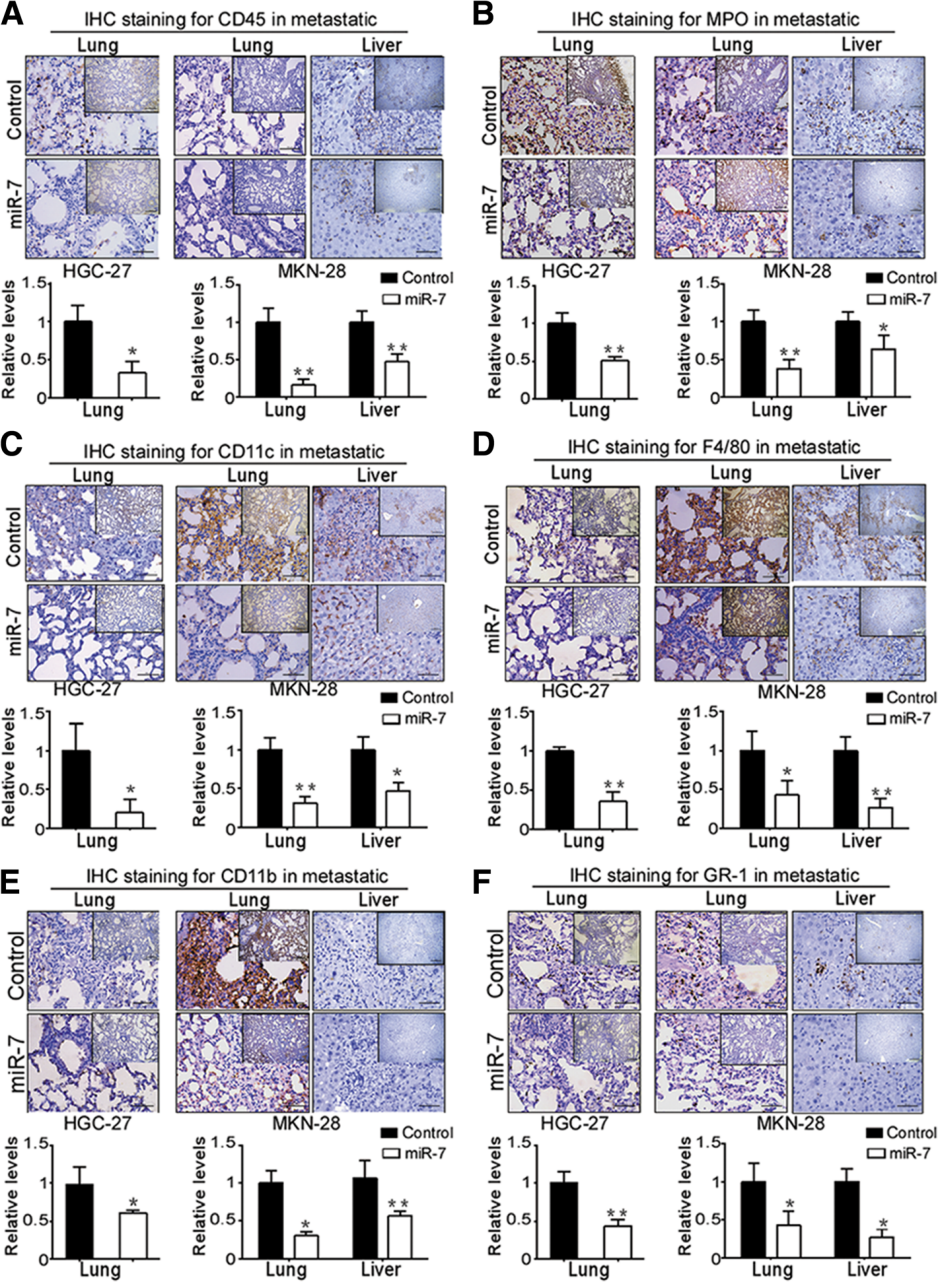
miR-7 alleviates inflammation cells infiltration in GC metastasis. Experimental metastasis mice were established as described in Materials & Methods. Inflammation cells was evaluated in embedded metastatic lung and liver tissues by IHC staining using specific antibodies including CD45 (**a**), MPO (**b**), CD11c (**c**), F4/80 (**d**), CD11b (**e**) and GR-1 (**f**). Positive staining cells were counted from 5 high magnification fields (× 400) in metastatic tissues. Relative expression was normalized to 1 compared with control group. Representative IHC images are shown. *p < 0.05, **p < 0.01 and ***p < 0.001 between the indicated two groups determined by paired student’s *t* test. Scale bars: (main) 50 μm; (inset) 200 μm

Inflammation can fuel both primary tumor growth and metastasis and has been long recognized as a key aspect of cancer development [19, 20, 23]. To determine whether inflammatory cells are involved in miR-7-mediated metastatic inhibition, using T and B lymphocytes-deficient nude mice model, we analyzed the expression of inflammatory cells marker such as the pan-leukocyte marker CD45, the neutrophil marker MPO, the macrophage markers CD11b, F4/80 and other markers GR1, CD11c in metastatic tissues via IHC staining. We found that CD45 and MPO positive cells in metastatic tissues were significantly decreased in miR-7-transfected group compared with control group (Fig. 7a-b). Subsequently, we also found a significant decreased intensity of CD11c, F4/80, CD11b and Gr-1-positive cells in metastatic lung and liver tissues of miR-7 transfected group compared with control group (Fig. 7c-f). These results indicated that miR-7 could improve infiltrating immune cells-mediated inflammation in GC.

### p65-mediated NF-κB activation is critical to anti-metastasis activation of miR-7 in GC

**Fig. 8:**
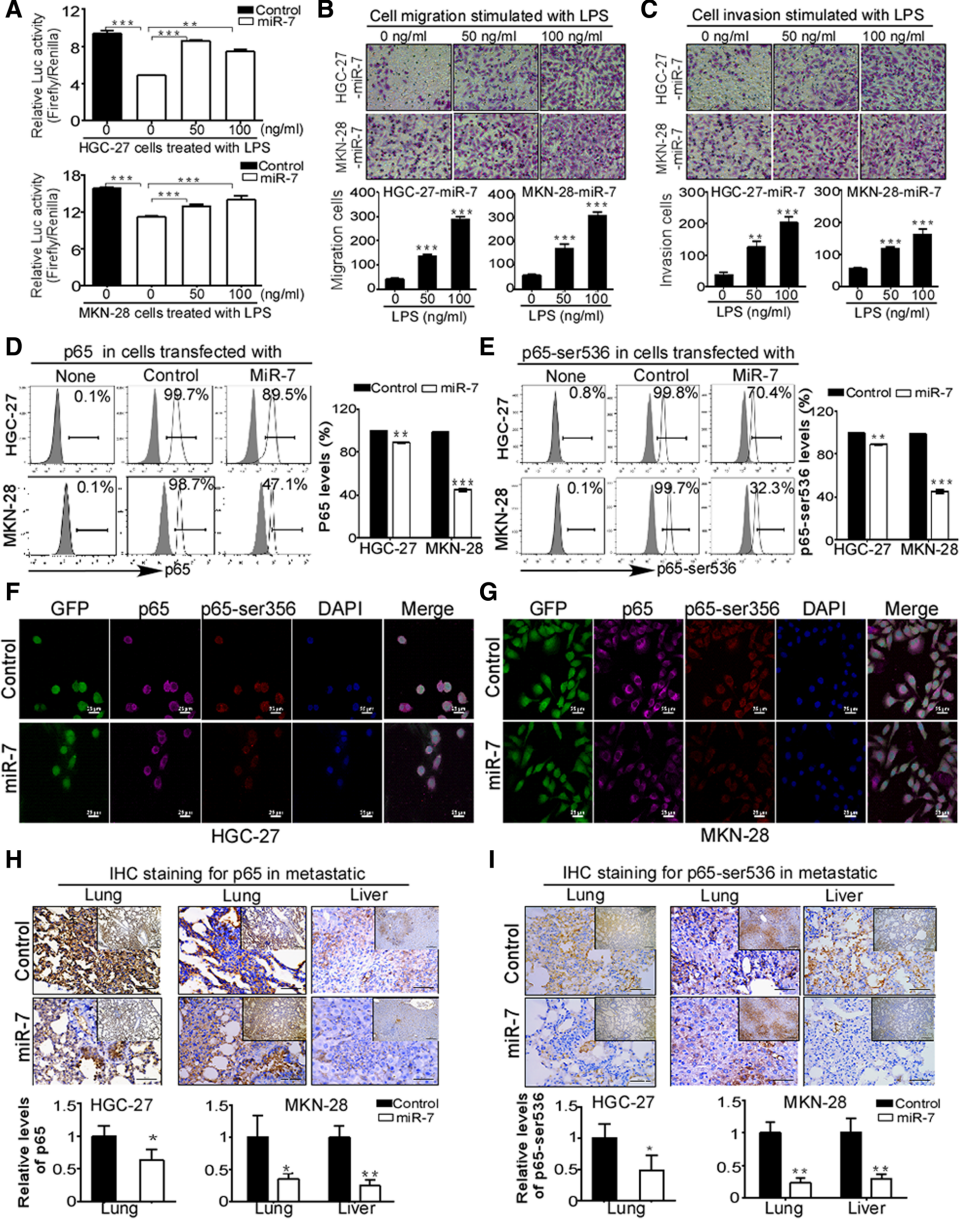
miR-7 suppresses NF-κB transcriptional activity by reducing p65 and active NF-κB/p65 expression. **a** miR-7 suppressed NF-κB transcriptional activity while NF-κB activator LPS reversed this activity. NF-κB transcriptional activity was detected by dual-luciferase reporter assay. NF-κB activator LPS was used in indicated groups. The data were presented as fold inductions of the ratio was normalized to *Renilla* luciferase activity. **b-c** LPS treatment reverted the abilities of cell migration and cell invasion after miR-7 transfection. Indicated cells were treated with or without 50 ng/ul or 100 ng/ul LPS and then cell migration and cell invasion were analyzed in miR-7-transfected HGC-27 (**b**) and MKN-28 cells (**c**). Data are presented as mean ± SD of three independent experiments. Representative images are shown. **d-e** Restoration of miR-7 decreased total p65 and active p-p65(Ser536) levels in GC cells. p65 and p-p65(Ser536) proteins were detected by FACS analysis in miR-7-transfected HGC-27 (**d**) and MKN-28 cells (**e**). Representative images are shown. **f-g** Cellular distribution of p65 and p-p65(Ser536) in miR-7-transfected GC cells. Immunofluorescence (IF) staining was used to detect cellular distribution of p65 and p-p65(Ser536) in indicated GC cells (GFP). AF555 (Red) or AF647 (Pink)-conjugated secondary antibodies were used to detect primary antibodies. Nuclei were counterstained with DAPI (Blue). Images were captured using a confocal microscope (Scale bars: 25 μm). **h-i** Restoration of miR-7 reduced p65 and p-p65(Ser536) expression in metastatic tissues. p65 and p-p65(Ser536) were detected by IHC staining in metastatic lung and liver tissues. Representative IHC images are shown. Scale bars: (main) 50 μm; (inset) 200 μm. *p < 0.05, **p < 0.01, ***p < 0.001 between the indicated two groups determined by paired student’s *t* test

Considering the fact that multiple target genes of miR-7 and diverse regulatory miRNAs for RelA/p65, we want to know whether NF-κB activity is critical to miR-7-counteracted metastasis in GC cells. Using NF-κB luciferase reporter assay, we found that miR-7 remarkably attenuated NF-κB transcriptional activity while these inhibitions were significantly reverted upon NF-κB activator LPS stimulation in HGC-27 and MKN-28 cells (Fig. 8a), indicating miR-7 negatively regulates NF-κB transcriptional activity. To demonstrate the essential role of NF-κB activation in anti-metastasis activation of miR-7 in vitro, we further test whether LPS-induced restoration of NF-κB activity could reverse miR-7-inhibited metastasis in vitro. As we expected, LPS treatment markedly restored the abilities of cell migration and invasion with dose dependent manner in miR-7-transfected HGC-27 and MKN-28 cells (Fig. 8b-c). Moreover, we also examined NF-κB downstream metastasis-related targets levels including Vimentin, ICAM-1, VCAM-1, MMP-2 and MMP-9 through flow cytometry analysis and IHC staining. Our finding showed that miR-7-transfection significantly decreased these downstream targets expression in GC cells (Additional file 8: Figure S6A-B) and metastatic tissues (Additional file 8: FigureS6C-E), respectively. These results suggested that NF-κB transcriptional activation is critical to anti-metastasis activation of miR-7.

To determine the details for the regulation of miR-7 to NF-κB activation, we further analyzed the levels of NF-κB subunits by FACS analysis in vitro. Interestingly, miR-7 transfection did not alter p50 and p52 expressions (data not shown) but significantly decreased levels of total p65 and p-p65(ser536) (a surrogate marker for NF-κB activity) in HGC-27 and MKN-28 cells (Fig. 8d-e). Moreover, IF analysis showed that miR-7 obviously decreased both cytoplasmic and nuclear distribution of p65 and p-p65(ser536) in GC cells (Fig. 8f-g), Meanwhile, we did not observe typical nuclear export of p65 in these cells. In addition, decreased p65 and p-p65(ser536) expressions were also confirmed by IHC staining in metastatic tissues (Fig. 8h-i). indicating that decreased NF-κB/p65 expression should be responsible for the miR-7-inhibited NF-κB activation. Together, these data suggest that miR-7 negatively controls NF-κB transcriptional activity and its downstream metastasis-associated targets expression by decreasing NF-κB/p65 activation and thereby inhibits GC metastasis.

## Discussion

Identifying the critical mediators causing aberrant NF-κB activation may provide a new insight to understand the mechanisms of GC progression and shed light on potential diagnostic targets and therapeutic strategies for GC metastasis. The dysregulated profile of miR-7 and its tumor-suppressive role have been reported in many human cancers, however, emerging studies also revealed the opposite effect of miR-7 in tumorigenesis [12, 24, 25]. Emerging evidences also indicated that miR-7 knockdown facilitated tumor growth in some GC cell lines [12, 26]. However, the expression profile and clinical relevance of miR-7 as well as the mechanism underlying dysregulated miR-7 in human GC is yet to be elucidated. Notably, much less information is available for the therapeutic effect of miR-7 in GC metastasis. It is therefore of interest in our current study to mainly focus on the potentially therapeutic application of delivering miR-7 in GC metastasis and its possible mechanisms. In this study, by larger GC cohort analysis from the TCGA STAD and NCBI GEO database, we revealed expression profile of low miR-7 and high RelA/p65 in GC as well as a negative correlation of miR-7 with RelA/p65 expression in GC patients. Meanwhile, we further extended this analysis and revealed that miR-7 expression is negatively correlated with NF-κB downstream metastasis-related targets Vimentin, ICAM-1, VCAM-1, MMP-2, MMP-9 and VEGF mRNA levels. More significantly, our data further uncovered a clinical relevance of low miR-7 and high RelA/p65 as prognostic indicators of poor OS, FPS, PPS, PFS and DMFS in GC patients. These results strongly suggest that low miR-7 may contribute to aberrant NF-κB activation by regulating RelA/p65. To the best of our knowledge, this study is the first to define the prognostic significance of miR-7 and RelA/p65 for GC progression, providing a possible link between miR-7 loss and aberrant NF-κB activation in GC.

Our finding describes a novel mechanism for downregulated miR-7 of human GC cells involving in the impaired mature processing in miR-7 biogenesis, providing new avenues to develop novel therapeutic strategies for GC by regulating miR-7 biogenesis. Our study showed that downregulated miR-7 in human GC cells with a mechanism involving the mature processing defect of miR-7 biogenesis from three pre-miR-7 isoforms to mature miR-7. In support of our finding, a previous study from human glioblastomas also showed a processing defect from pri-miR-7 s to pre-miR-7 s in miR-7 biogenesis, leading to decreased mature miR-7 [7]. Therefore, the processing defect of miR-7 biogenesis directly or indirectly downregulate miR-7 expression through modulation of these regulation systems. However, it is still unclear what contributes to the impaired miR-7 mature processing in GC progression. It has been shown that dysregulated miRNAs are associated with genomic/epigenetic alterations or transcriptional/post-transcriptional mechanisms [25]. However, neither genomic deletion nor epigenetic mechanisms are responsible for the downregulation of miR-7 in GC cells [12], implying that other mechanisms were responsible. In fact, emerging studies reported possible mechanisms for dysregulated miR-7 in human cancers. For examples, miR-7 transcription can be directly induced by HoxD10 [27] or c-Myc [28]. Splicing factor SF2/ASF also can bind the pri-miR-7 to enhance its cleavage by Drosha [29]. However, these evidences are not enough to provide a reasonable explanation for aberrant miR-7 in GC. Here, our finding showed dysregulated Dicer1 expressions in GC cells and further discovered an impaired pre-miR-7 processing and binding activities. Therefore, dysregulated Dicer1 enzymes impairing pre-miR-7 processing activity should be responsible for the downregulation of miR-7 expression in GC cells.

Our findings also offer a potential therapeutic strategy by delivering miR-7 for aberrant NF-κB-driven GC metastasis. Distant liver and lung metastasis are the most frequent forms of advanced GC metastasis [2]. Our data from in vitro and in vivo models revealed that lentivirus-mediated delivery of miR-7 (only 2 folds upregulation) exhibited delightful effects in controlling GC lung and liver metastasis and effectively improved OS in experimental metastasis mice models. Tumor Metastasis involves a complex series of processes including migration, adhesion, invasion via the bloodstream, via the lymphatic system, or by direct extension and even immune cells infiltrating [22, 30]. Our study demonstrated that miR-7 also controlled hemangiogenesis, lymphangiogenesis and inflammation cells infiltration, indicating that miR-7 possesses ability of targeting multiple metastatic processes. Therefore, delivery of miR-7 is encouraging and may represent an appealing approach for therapeutic effects to aberrant NF-κB-driven GC distant metastasis. In addition, our study also showed that cell cycle arrest but not cell apoptosis was involved in the suppressive role of miR-7 in cell viability, which is different with others reports that miR-7 induces cell apoptosis in GC cells [12]. Therefore, further study is need to demonstrate the context dependent difference of miR-7 in GC tumorigenesis.

Our findings also reveal that p65-mediated NF-κB activation is critical to anti-metastasis activation of miR-7 in GC, providing a reasonable explanation for the anti-metastasis ability of miR-7. One of the most appealing properties of miRNAs is their abilities to target multiple genes in regulating distinct biological processes [3]. Previous studies identified a range of potential targets of miR-7 including EGFR, IRS1/2, IGF1R, PAK-1, RAF-1 and SATB1 [11, 12, 24, 31]. Recent emerging evidences also indicated that miR-7 regulated RelA/p65 expression via directly binding 3’-UTR of RelA/p65 mRNA in some GC cell lines [12, 26]. However, GC metastasis is typically not driven by aberrant EGFR, IRS1/2, IGF1R expression and/ or signaling though these targets have been shown to be related to GC progression [12, 24, 31]. Therefore, the priority of our studies is to focus on the critical role of NF-κB activation in anti-metastasis activation of miR-7 in GC. Here, our studies revealed that miR-7 inhibited NF-κB transcription activity and decreased NF-κB downstream metastasis-related proteins by regulating NF-κB p65 and active p-p65-ser536 levels rather than p50 and p52 expression. Moreover, using pharmacologic prevention strategy with NF-κB activator, we revealed LPS stimulation obviously restored miR-7-suppressed NF-κB transcriptional activation and significantly reverted miR-7-inhibited cell migration and invasion, indicating an essential role of NF-κB activation in miR-7-mediated anti-metastasis activation. Therefore, it is conceivable that dysregulated miR-7 lost the control to NF-κB/p65 levels, thereby facilitating aberrant NF-κB-driven GC distant metastasis and ultimately resulting in a poor clinical outcome.

Of note, our findings show that miR-7 improves inflammation cells infiltration, further providing a prospective therapeutic strategy by combination of miR-7 delivery and immunotherapies for GC metastasis treatment. Aberrant accumulation of inflammation cells such as neutrophils and macrophages are often associated with poor clinical outcomes and increased risk for tumor metastasis [20, 23, 32]. Meanwhile, recruitment of inflammation cells plays a crucial role in inducing inflammatory neovascularization by supplying/amplifying signals essential for pathological angiogenesis and lymphangiogenesis [18]. Noteworthy, among miR-7-regulated NF-κB downstream metastasis-related proteins, VEGF can induce inflammatory neovascularization for pathological hemangiogenesis and lymphangiogenesis by recruiting inflammation monocytes and (or) macrophages [18, 23]. Meanwhile, adhesion proteins ICAM-1 and VCAM-1 expressed on endothelium, as ligands by LFA-1 and Mac-1 (CD11b) expressed in leukocytes, play a crucial role in recruitment of circulating leukocytes into the inflammation sites [20, 23]. Our previous study showed that CD4^+^ and CD8^+^ T lymphocytes play crucial roles in breast cancer progression and outcome [21]. Here, using T and B lymphocytes-lacking nude mice, we found that MPO^+^, CD11b^+^, Gr-1^+^, F4/80^+^ and even CD11c^+^ immune cells of non-T and B lymphocytes are all involved in the metastasis -suppressive function of miR-7 in GC, providing the better understanding for the immune regulatory function of miR-7 in improving inflammation cells infiltration by reducing inflammation cytokines. Therefore, combination strategy of miR-7 delivery and immunotherapies may yield synergistic effects in treatment for GC metastasis.

## Conclusions

In summary, we demonstrated that low miR-7 was correlated with high RelA/p65 expression in GC and revealed a clinical relevance that low miR-7 and high RelA/p65 as prognostic indicators of worse clinical outcome in GC patients. Moreover, we highlight that an impaired miR-7 mature processing is associated with downregulation of miR-7 in GC cells. Delivering miR-7 displays therapeutic effect to GC distant metastasis by reducing NF-κB downstream metastasis-related molecules via inhibiting RelA/p65-mediated NF-κB activation. These studies suggest that miR-7 might be novel prognostic biomarker and potential therapeutic target for GC distant metastasis.

## Additional files

Additional file 1: **Table S1.** Primers for Real-time PCR in this study. **Table S2.** Antibodies information used in this study. **Table S3.** Primers of nest PCR for pre-MiR-7-1 DNA template. (DOC 66 kb)

Additional file 2: Supplemental Materials and Methods. (DOC 58 kb)

Additional file 3: **Figure S1.** Increased mRNA expression of metastasis-related NF-kB downstream genes in GC. The mRNA expression of NF-kB downstream and metastasis-related genes in GC was analyzed from the NCBI GEO database (Normal: normal gastric mucosa; Cancer: gastric cancer). (A) Vimentin mRNA expression of GC from NCBI/GEO/ GSE26942. (B) MMP-2 mRNA expression (Left panel, *P* < 0.001) and MMP-9 mRNA expression (Right panel, *P* < 0.01) from NCBI/GEO/GSE26942. (C) VEGF mRNA expression from NCBI/GEO/GSE26942. (D) The mRNA expression of ICAM-1 (Left panel, *P* < 0.01) and VCAM-1(Right panel, *P* < 0.001) according to NCBI/GEO/GSE26942. **p < 0.01, ***p < 0.001 between the indicated two groups determined by paired student’s *t* test. (TIF 799 kb)

Additional file 4: **Figure S2.** Real-time PCR for mRNA expression of HNRPK, PGSF1, DROSHA and DICER1 in GC cell lines. Total RNA was extracted from indicated cells. Real-time PCR was performed to detect mRNA expressions of HNRPK, PGSF1 (A) and DROSHA, DICER1 (B). β-actin was used as an internal control. Data are presented as mean ± SD. **p < 0.01, ***p < 0.001 compared with GES-1 group determined by paired student’s *t* test. (TIF 824 kb)

Additional file 5: **Figure S3.** Cloning and transcription of pre-miR-7-1. Schematic illustration of the cloning and in vitro transcription of Pre-miR-7-1 (A). Outer primers pairs 483F/483R was used to amplify 483-bp Pre-miR-7-1 DNA fragments from the genomic DNA (NC_000009.12). Inner primers pairs 130F/ T7–130R was used to obtain 130-bp pre-miR-7-1 transcriptional templates containing a complementary T7 promoter sequence downstream of the RNA coding sequences.110-nt pre-miR-7-1 RNA was obtained by In vitro transcription and biotin labeling using T7 run-off primers. Stem-loop pre-miR-7-1 RNA was obtained by in vitro RNA folding. 483bp and 130 bp PCR products were analyzed by agarose gel electrophoresis (B). DNA sequence was confirmed by DNA sequencing (C). (TIF 203 kb)

Additional file 6: **Figure S4.** Lentivirus-mediated mature miR-7 expression in GC cells. Lentivirus-mediated mature miR-7 and control miRNA (control) were transfected into HGC-27 and MKN-28 GC cells. Post-transfection, GFP^+^ infected cells were sorted by FACS (A) and were then expanded in vitro (B), original magnification: × 100. miR-7 expression was detected by real-time PCR in indicated cells (C). U6 RNA was used as internal control. **p < 0.01, ***p < 0.001 between the indicated two groups determined by paired student’s *t* test. (TIF 794 kb)

Additional file 7: **Figure S5.** Immunofluorescence analysis for Ki67 expression in miR-7-transfected GC cells. Immunofluorescence (IF) analysis was performed to detect Ki67 expression in HGC-27 cells (A) and MKN-28 cells (B). Indicated cells were transfected with miR-7 or control lentivirus (GFP, green) and ki67 expression was analyzed with primary ki67 antibodies and AF555-conjugated secondary antibody (Red). Nuclei were counterstained with DAPI (Blue). Images were captured using a confocal microscope (Scale bars: 200 μm). Representative IF images are shown. (TIF 1107 kb)

Additional file 8: **Figure S6.** miR-7 suppresses NF-κB downstream metastatic genes expression in vitro and in vivo. (A) Restoration of of miR-7 inhibited the expression of NF-κB downstream metastatic genes expression in HGC-27 cells. HGC-27 were stably transfected with miR-7 and control. NF-κB downstream targets including Vimentin, ICAM-1, VCAM-1, MMP-2, MMP-9, VEGF and were detected by FACS analysis. Representative FACS images are shown. (B) Restoration of of miR-7 inhibited the expression of NF-κB downstream metastatic genes expression in MKN-28 cells in vitro. MKN-28 were stably transfected with miR-7 and control. NF-κB downstream targets including Vimentin, ICAM-1, VCAM-1, MMP-2, MMP-9 and VEGF were detected by FACS analysis. Representative FACS images are shown. (C-E) Ectopic expression of miR-7 markedly suppressed NF-κB-responsive targets in metastatic tissues of HGC-27 cells. NF-κB-responsive targets including Vimentin, ICAM-1, VCAM-1, MMP-2, MMP-9 and VEGF were measured using IHC staining in metastatic lung of HGC-27 cells(C), metastatic lung (D) and liver (E) tissues of MKN-28 cells. Representative IHC images are shown. *p < 0.05, **p < 0.01 between the indicated two groups determined by paired student’s *t* test. Scale bars: (main) 50 μm; (inset) 200 μm. (TIF 3696 kb)

## Abbreviations

DMFS: Distant metastasis-free survival
FPS: First progression survival
GC: Gastric cancer
GRIM-19: Gene associated with retinoid-IFN-induced mortality 19
ICAM-1: Intercellular cell adhesion molecule-1
MPO: Myeloperoxidase
OS: Overall survival
PFS: Progression-free survival
PPS: Post progression survival
VCAM-1: Vascular cell adhesion molecule-1
