# Supplementary material for: MicroRNA-7 as a potential therapeutic target for aberrant NF-κB-driven distant metastasis of gastric cancer
Source: J Exp Clin Cancer Res. 2019 Feb 6;38:55. doi: 10.1186/s13046-019-1074-6 (PMC6364399; doi:10.1186/s13046-019-1074-6)
Supplement: Supplementary file 1 — Table S1. Primers for Real-time PCR in this study. Table S2. Antibodies information used in this study. Table S3. Primers of nest PCR for pre-MiR-7-1 DNA template. (DOC 66 kb) [file 13046_2019_1074_MOESM1_ESM.doc]

**Additional file Tables**

**Table S1** Primers for Real-time PCR in this study

| **Gene (GenBank**  **Accession No)** | **Primer sequences**  **(5'-3')** | **Annealing**  **Temp (℃)** | **Size**  **(bp)** |
| --- | --- | --- | --- |
| VEGF-C  (NM_ 005429.4) | F: CTACCACAGTGTCAGGCAGC  R: TCTTCCTGAGCCAGGCATCT | 63 | 86 |
| VEGF-A  (NM_001025366.2) | F: AGGGCAGAATCATCACGAAGT  R: AGGGTCTCGATTGGATGGCA | 63 | 75 |
| HNRPK: (NM_001318187.1) | F: CCTGCGCTCGTTTTCTGTCT  R: AACGGGCACACCAATCAGTT | 60 | 167 |
| PGSF1  (NR_027148.1) | F: GCGAAGTTTGACGGTCTGGA  R:GAGTGCATTCCAAGCTGGAGG | 60 | 195 |
| Drosha  NM_001100412.1 | F: ATCTGGAAGTCGCTCCCCAA  R: TCTGGTTGTCACTCCAACGG | 60 | 80 |
| Dicer1  NM_001291628.1 | F: TCGAGCCTCCATTGTTGGTC  R: TGGTCATCCAGTTCGCCAAT | 60 | 108 |
| β-ACTIN  (NM_001101.4) | F: CGGCGCCCTATAAAACCCA  R: ATCATCCATGGTGAGCTGGC | 60-63 | 130 |

F: Forward; R: Reverse;

**Table S2 Antibodies information used in this study**

| **Antibodies** | **Catalog number** | | **Dilution**  **for FACS** | **Dilution**  **for IHC** | **Dilution**  **for IF** |
| --- | --- | --- | --- | --- | --- |
| p65 | Zsbio Inc | zs-8008 | 1:50 | 1:800 | 1:200 |
| p-p65(ser536) | Santa Cruz | sc-101752 | 1:50 | 1:800 | 1:200 |
| Ki67 | Abcam | ab15580 | 1:100 | / | 1:200 |
| VEGF | Bioss Inc | bs-0279R | 1:200 | 1:800 | / |
| MMP2 | Bioss Inc | bs-0412R | 1:200 | 1:800 | / |
| MMP9 | Bioss Inc | bs-4593R | 1:200 | 1:800 | / |
| ICAM-1 | Bioss Inc | bs-4618R | 1:200 | 1:800 | / |
| VCAM-1 | Bioss Inc | bs-0396R | 1:200 | 1:800 | / |
| Vimentin | Bioss Inc | bs-0756R | 1:200 | 1:800 | / |
| LYVE-1 | Bioss Inc | bs-20353R | / | 1:1000 | / |
| MPO | Boster Biotech | PB0072 | / | 1:400 | / |
| CD34 | Biolegend | 128601 | / | 1:200 | / |
| CD45 | Biolegend | 103101 | / | 1:200 | / |
| CD11b | Biolegend | 101201 | / | 1:100 | / |
| CD11c | Biolegend | 117301 | / | 1:200 | / |
| F4/80 | Biolegend | 123108 | / | 1:200 | / |
| GR-1 | Biolegend | 108401 | / | 1:200 | / |

Note: Zsbio Inc: Beijing Zhongshang Golden Bridge Co., Ltd;

Bioss Inc: Beijing Biosynthesis Biotechnology Co., Ltd.

**Table S3** Primers of nest PCR for pre-MiR-7-1 DNA template

| **Nest PCR** | **Primer sequences** **(5'-3')** | **Annealing**  **Temp (℃)** | **Size**  **(bp)** |
| --- | --- | --- | --- |
| 1^st^ round | 483F: CTACCACAGTGTCAGGCAGC  483R: TCTTCCTGAGCCAGGCATCT | 61 | 483 |
| 2^nd^ round | 130F: TTGGATGTTGGCCTAGTTCTGTGTGGA  T7-130R: TAATACGACTCACTATAGGGCTGTAGAGGCATGGC | 65 | 130 |

Note: Underline indicates T7 promoter sequence; F: Forward; R: Reverse;
