# Supplementary material for: MicroRNA-7 as a potential therapeutic target for aberrant NF-κB-driven distant metastasis of gastric cancer
Source: J Exp Clin Cancer Res. 2019 Feb 6;38:55. doi: 10.1186/s13046-019-1074-6 (PMC6364399; doi:10.1186/s13046-019-1074-6)
Supplement: Supplementary file 2 — Supplemental Materials and Methods. (DOC 58 kb) [file 13046_2019_1074_MOESM2_ESM.doc]

**Supplemental Materials and Methods**

**1. In vitro transcription and labeling of pre-miR-7-1**

**1.1** **Cloning of transcriptional PCR template of pre-miR-7-1 DNA**

To create pre-MiR-7-1 transcription RNA substrate (MI0000263), PCR templates of pre-miR-7-1 containing a complementary T7 promoter sequence upstream of the RNA coding sequences were obtained by amplification of the genomic DNA of GES-1 cells by nest PCR using Plantium Taq DNA polymerase (Invitrogen, USA). A 483-bp DNA fragment containing pre-miR-7-1 DNA was amplificated by 1st round PCR from the genomic DNA (NC_000009.12) of human GES-1 cells using primers pairs 483F/483R. After 2^nd^ round PCR, a 130-bp PCR fragments with T7 promoter sequence (5'-TAATACGACTCACTATAGGG-3') were obtained with 483-bp DNA fragment as PCR template using primers pairs 130F/T7-130R (The details of primers are shown in supplemental Table S3). PCR products was confirmed by DNA sequencing.

**1.2 In vitro transcription and labeling of pre-MiR-7-1**

To synthesize RNAs, 130bp pre-miR-7-1 PCR templates were used to synthesize 110 nt pre-miR-7-1 RNA by T7 RNA polymerase in vitro transcription using T7 run-off primers. In vitro transcription, biotin body-labeling and purification of pre-miR-7-1 RNA were performed using a Ribo^TM^RNAmax-T7 transcription and biotin RNA labeling Kit (RIOBOBIO, Guangzhou, China) according to the manufacturer’s instructions. Biotin-labeled pre-miR-7-1 transcriptions were diluted to 100nM in TE buffer and heated at 95°C for 3 min and then slowly cooled to 25°C for RNA folding. 2-2.5nM aliquots Pre-miR-7-1 were made and stored at -80°C.

**2. Preparation of Cell extracts and Dicer1 containing IP complex**

Cell extracts from different cells lines were prepared using Western and IP cell lysis buffer (Beyotime, Haimen, China) according to the manufacturer’s instructions. Cell extracts were centrifuged at 4°C,13000 rpm for 5 min and the extract supernatants were collected. Protein concentration was determined by BCA kit (Beyotime, Haimen, China). Extracts concentration were subsequently adjusted to approximately 5-10 µg/µl using dilution buffer (100 mM KCl, 2 mM MgCl_2_ and 10% glycerol).

Dicer1 containing complex was immunoprecipitated from cell extracts using Protein A/G PLUS-Agarose (Santa Cruz, USA) and Rabbit anti-human Dicer1 antibody (Bioss, Beijing, China) according to the manufacturer’s instructions. Briefly, cell extracts (100ug) were prepared using Western and IP cell lysis buffer. After precleared using 1.0 µg normal Rabbit IgG and 20ul Protein A/G PLUS-Agarose at 4°C for 30minutes, cell supernatants were collected and incubated with 1µg Rabbit anti-Dicer1 antibody and 10µl Protein A/G PLUS-Agarose at 4°C for 2 hours. After centrifugation at 1,000g for 5min at 4°C, IP complexes were washed 4 times with 1.0 ml Western and IP cell lysis buffer and submitted to the further analysis.

**3. In vitro pre-MiR-7-1 processing analysis using cell extracts or Dicer1 containing IP complex**

Pre-miR-7-1 processing was performed using cell extracts or Dicer1 containing IP complex respectively. The processing reaction consisted of 40 µg indicated cell extracts (or 0.4µg anti-Dicer1 immunoprecipitated Dicer1 containing complex), 2 nM pre-miR-7-1 substrate and 1 unit/μl Ribonuclease inhibitor (Takara) in a final volume of 20µl processing buffer (20 mM Tris-HCl, pH 7.9, 0.1 M KCl, 10% glycerol, 5 mM DTT, and 0.2 mM PMSF). The processing reaction mixture was incubated at 37°C for 60 min and was loaded on 15% denaturing PAGE gels, transferred to Nylon N^+^ membranes (Chleicher & Schuell) and crosslinked 3-5 min by 254nm UV lamp. The biotin-labeled 110 nt pre-miR-7-1 and mature ~21-23nt miR-7 were submitted to RNA gel blot analysis using LightShift Chemiluminescent RNA EMSA Kit (Thermo Fisher Scientific, MA, USA) according to the manufacturer’s instructions. The biotin-labeled 28nt RNA was used as a loading control.

**4. In vitro pre-MiR-7-1 binding analysis using cell extracts or Dicer1 containing IP complex**

Pre-MiR-7-1 binding analysis was performed using 4 ug cell extracts (or 0.1µg anti-Dicer1 immunoprecipitated Dicer1 containing complex) and 5 nM pre-miR-7-1 RNA substrate at 25°C for 30 min in binding reaction buffer by LightShift Chemiluminescent RNA EMSA Kit according to the manufacturer’s instructions. The protein/pre-miR-7-1 binding complex was loaded and resolved on 5% denaturing PAGE gels, transferred to Nylon N^+^ membranes (Chleicher & Schuell) and crosslinked 3-5 min by 254nm UV lamp. The biotin-labeled shift binding or free pre-miR-7-1 were detected by LightShift Chemiluminescent RNA EMSA Kit (Thermo Fisher Scientific, MA, USA) according to the manufacturer’s instructions.

**References**:

1. Leuschner P J F, Martinez J. In vitro analysis of microRNA processing using recombinant Dicer and cytoplasmic extracts of HeLa cells. Methods (Amsterdam), 2007, 43(2):0-109.

2. Kumar S, Velasco A D R, Michlewski G. Oleic Acid Induces MiR-7 Processing through Remodeling of Pri-MiR-7/Protein Complex. Journal of Molecular Biology, 2017, 429(11):1638-1649.
