# Supplementary figures and images for: MicroRNA-7 as a potential therapeutic target for aberrant NF-κB-driven distant metastasis of gastric cancer
Source: J Exp Clin Cancer Res. 2019 Feb 6;38:55. doi: 10.1186/s13046-019-1074-6 (PMC6364399; doi:10.1186/s13046-019-1074-6)

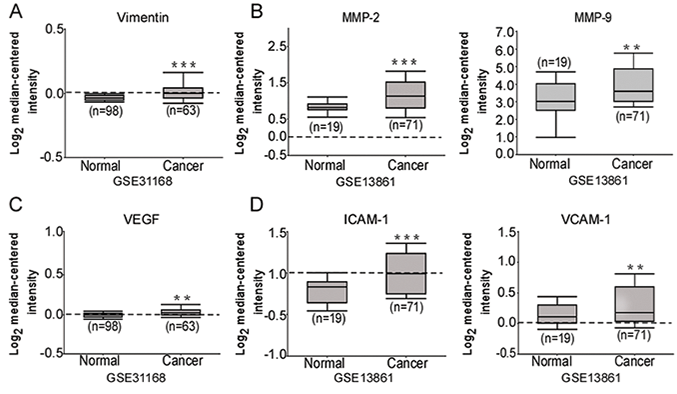

Supplement: Supplementary file 3 — Figure S1. Increased mRNA expression of metastasis-related NF-kB downstream genes in GC. The mRNA expression of NF-kB downstream and metastasis-related genes in GC was analyzed from the NCBI GEO database (Normal: normal gastric mucosa; Cancer: gastric cancer). (A) Vimentin mRNA expression of GC from NCBI/GEO/ GSE26942. (B) MMP-2 mRNA expression (Left panel, P < 0.001) and MMP-9 mRNA expression (Right panel, P < 0.01) from NCBI/GEO/GSE26942. (C) VEGF mRNA expression from NCBI/GEO/GSE26942. (D) The mRNA expression of ICAM-1 (Left panel, P < 0.01) and VCAM-1(Right panel, P < 0.001) according to NCBI/GEO/GSE26942. **p < 0.01, ***p < 0.001 between the indicated two groups determined by paired student’s t test. (TIF 799 kb) [file 13046_2019_1074_MOESM3_ESM.tif]

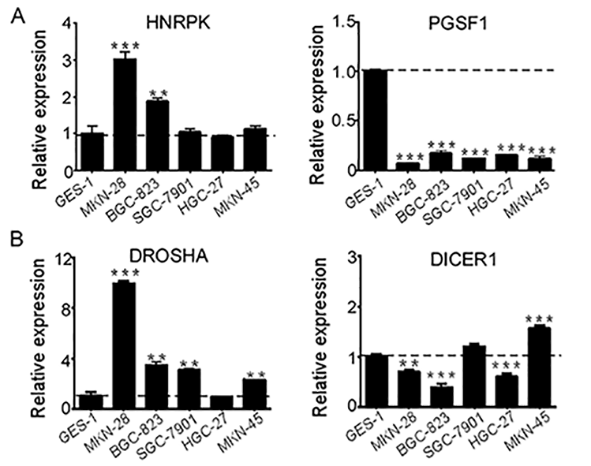

Supplement: Supplementary file 4 — Figure S2. Real-time PCR for mRNA expression of HNRPK, PGSF1, DROSHA and DICER1 in GC cell lines. Total RNA was extracted from indicated cells. Real-time PCR was performed to detect mRNA expressions of HNRPK, PGSF1 (A) and DROSHA, DICER1 (B). β-actin was used as an internal control. Data are presented as mean ± SD. **p < 0.01, ***p < 0.001 compared with GES-1 group determined by paired student’s t test. (TIF 824 kb) [file 13046_2019_1074_MOESM4_ESM.tif]

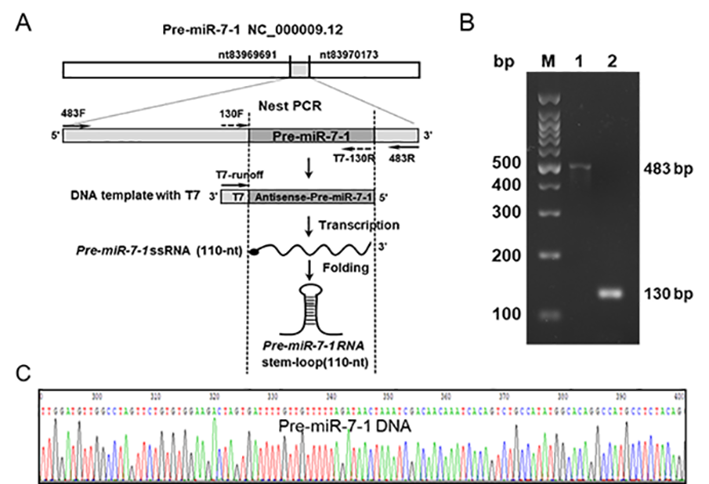

Supplement: Supplementary file 5 — Figure S3. Cloning and transcription of pre-miR-7-1. Schematic illustration of the cloning and in vitro transcription of Pre-miR-7-1 (A). Outer primers pairs 483F/483R was used to amplify 483-bp Pre-miR-7-1 DNA fragments from the genomic DNA (NC_000009.12). Inner primers pairs 130F/ T7–130R was used to obtain 130-bp pre-miR-7-1 transcriptional templates containing a complementary T7 promoter sequence downstream of the RNA coding sequences.110-nt pre-miR-7-1 RNA was obtained by In vitro transcription and biotin labeling using T7 run-off primers. Stem-loop pre-miR-7-1 RNA was obtained by in vitro RNA folding. 483bp and 130 bp PCR products were analyzed by agarose gel electrophoresis (B). DNA sequence was confirmed by DNA sequencing (C). (TIF 203 kb) [file 13046_2019_1074_MOESM5_ESM.tif]

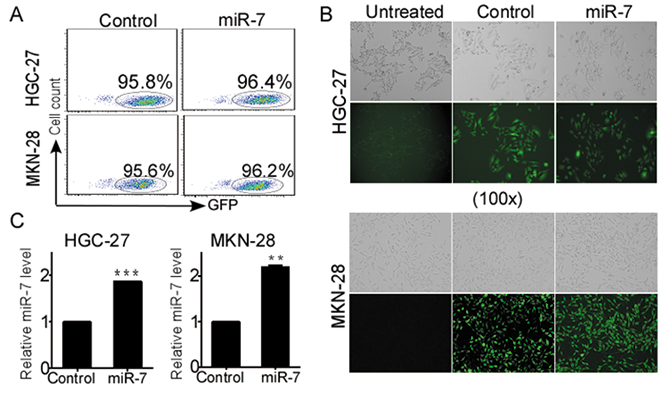

Supplement: Supplementary file 6 — Figure S4. Lentivirus-mediated mature miR-7 expression in GC cells. Lentivirus-mediated mature miR-7 and control miRNA (control) were transfected into HGC-27 and MKN-28 GC cells. Post-transfection, GFP+ infected cells were sorted by FACS (A) and were then expanded in vitro (B), original magnification: × 100. miR-7 expression was detected by real-time PCR in indicated cells (C). U6 RNA was used as internal control. **p < 0.01, ***p < 0.001 between the indicated two groups determined by paired student’s t test. (TIF 794 kb) [file 13046_2019_1074_MOESM6_ESM.tif]

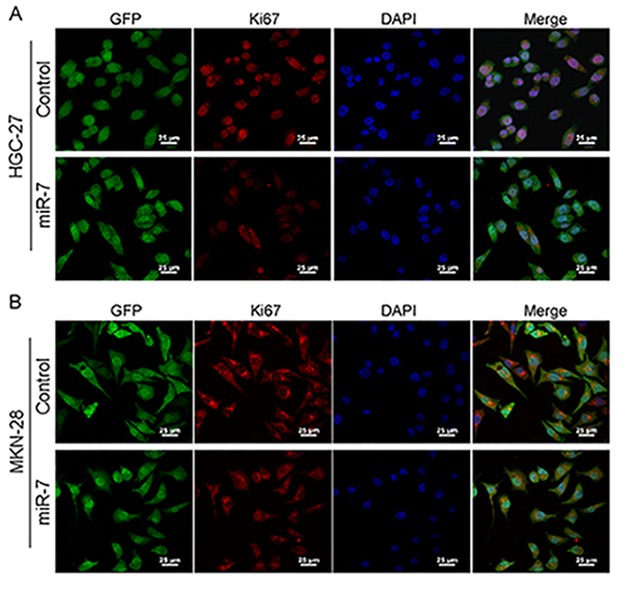

Supplement: Supplementary file 7 — Figure S5. Immunofluorescence analysis for Ki67 expression in miR-7-transfected GC cells. Immunofluorescence (IF) analysis was performed to detect Ki67 expression in HGC-27 cells (A) and MKN-28 cells (B). Indicated cells were transfected with miR-7 or control lentivirus (GFP, green) and ki67 expression was analyzed with primary ki67 antibodies and AF555-conjugated secondary antibody (Red). Nuclei were counterstained with DAPI (Blue). Images were captured using a confocal microscope (Scale bars: 200 μm). Representative IF images are shown. (TIF 1107 kb) [file 13046_2019_1074_MOESM7_ESM.tif]

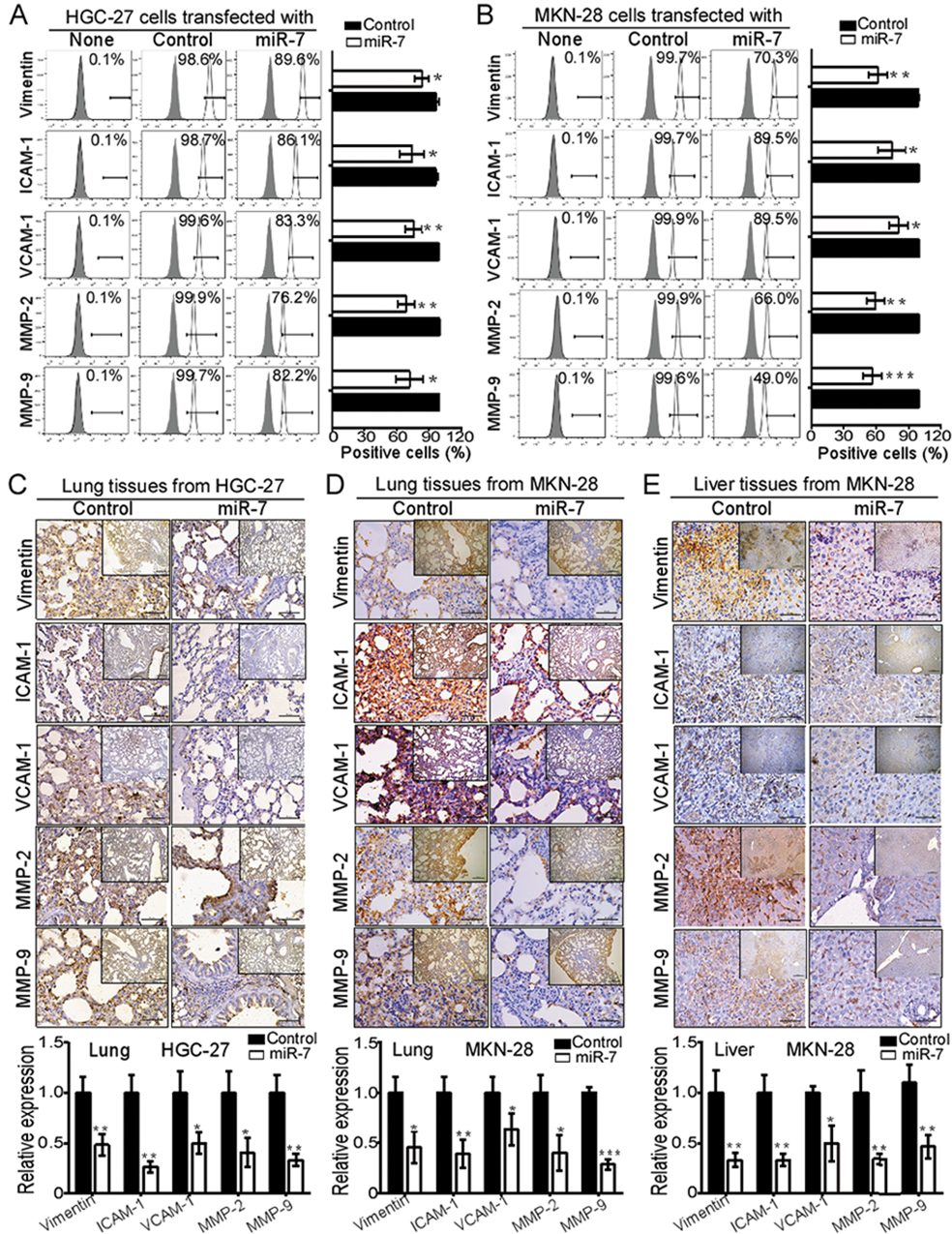

Supplement: Supplementary file 8 — Figure S6. miR-7 suppresses NF-κB downstream metastatic genes expression in vitro and in vivo. (A) Restoration of of miR-7 inhibited the expression of NF-κB downstream metastatic genes expression in HGC-27 cells. HGC-27 were stably transfected with miR-7 and control. NF-κB downstream targets including Vimentin, ICAM-1, VCAM-1, MMP-2, MMP-9, VEGF and were detected by FACS analysis. Representative FACS images are shown. (B) Restoration of of miR-7 inhibited the expression of NF-κB downstream metastatic genes expression in MKN-28 cells in vitro. MKN-28 were stably transfected with miR-7 and control. NF-κB downstream targets including Vimentin, ICAM-1, VCAM-1, MMP-2, MMP-9 and VEGF were detected by FACS analysis. Representative FACS images are shown. (C-E) Ectopic expression of miR-7 markedly suppressed NF-κB-responsive targets in metastatic tissues of HGC-27 cells. NF-κB-responsive targets including Vimentin, ICAM-1, VCAM-1, MMP-2, MMP-9 and VEGF were measured using IHC staining in metastatic lung of HGC-27 cells(C), metastatic lung (D) and liver (E) tissues of MKN-28 cells. Representative IHC images are shown. *p < 0.05, **p < 0.01 between the indicated two groups determined by paired student’s t test. Scale bars: (main) 50 μm; (inset) 200 μm. (TIF 3696 kb) [file 13046_2019_1074_MOESM8_ESM.tif]
